# Two zinc ABC transporters contribute to *Rhizobium leguminosarum* symbiosis with *Pisum sativum* and *Lens culinaris*

**DOI:** 10.3389/fpls.2025.1598744

**Published:** 2025-06-09

**Authors:** Joanna N. Soldek, Marta Ballesteros-Gutiérrez, Laura Díaz-Sáez, Ignacio Delgado-Santamaría, José Manuel Palacios, Marta Albareda

**Affiliations:** ^1^ Centro de Biotecnología y Genómica de Plantas, Universidad Politécnica de Madrid – Instituto Nacional de Investigación y Tecnología Agraria y Alimentaria (INIA/CSIC), Madrid, Spain; ^2^ Instituto de Química Física Blas Cabrera, Consejo Superior de Investigaciones Científicas (CSIC), Madrid, Spain; ^3^ Departamento de Biotecnología-Biología Vegetal, Escuela Técnica Superior de Ingeniería Agronómica, Alimentaria y de Biosistemas, Universidad Politécnica de Madrid, Madrid, Spain

**Keywords:** *Rhizobium leguminosarum*, nitrogen fixation, zinc uptake, symbiosis, legumes, pea, lentil

## Abstract

The establishment of the rhizobium-legume symbiosis requires adjusting the behavior of both partners to nodule conditions in which transition metals are delivered to the bacteria, as many rhizobial metalloenzymes are essential for bacteroid functions and symbiotic performance. A previous proteomic analysis revealed the existence of a relevant number of proteins differentially expressed in bacteroids induced by *Rhizobium leguminosarum* bv. viciae (Rlv) UPM791 in pea and lentil nodules. Among these proteins, a metal-binding protein (RLV_3444) component of an ABC-transporter system (RLV_3442-3444) was shown to be overexpressed in pea bacteroids, suggesting that metal provision to the bacteroid is more restrictive in the rhizobium-pea symbiosis. In this work, protein sequence analysis and structural modelling have revealed that RLV_3444 is highly similar to the functionally characterized zinc-binding protein ZniA from *Klebsiella pneumoniae*, so the host-dependent binding protein was renamed as ZniA and the transporter system as ZniCBA. The genome of Rlv UPM791 also encodes the conserved high-affinity ZnuABC transporter system. We demonstrate that at least one of the two systems must be present for Rlv to grow under zinc-limiting conditions and for optimal symbiotic performance with pea and lentil plants. The three conserved histidine residues present in multiple Zn^2+^-binding proteins have been shown as essential for the function of Rlv ZniA, and *in-silico* modelling suggests that they might participate in metal coordination. We also demonstrate that both ZniCBA and ZnuA are regulated by zinc in a Zur-dependent manner, consistent with the presence of a Zur box in their regulatory region. The expression patterns revealed that ZniCBA is expressed at lower levels than ZnuA, and its expression increased in a *znuA* mutant under both free-living and symbiotic conditions. These results, along with the observed increment in the expression of ZniCBA in pea *versus* lentil bacteroids, suggest that the host-dependent transporter system might play an auxiliary function for zinc uptake under zinc starvation conditions and might play a relevant role in the adaptation of rhizobia to the legume host.

## Introduction

Zinc is an essential nutrient for cells, as it is required for catalytic or structural functions in numerous proteins (Blindauer, 2015). Excess concentrations of zinc become toxic by competing with other metal ions for biologically important ligands, and so intracellular zinc levels must be tightly regulated. Bacterial zinc homeostasis requires the balance of import and export systems to control zinc concentration inside the cell, thus preventing metal toxicity (Choi and Bird, 2014). Under zinc-repleted conditions, zinc supply is mediated by low-affinity transporters such as the constitutively expressed ZupT, belonging to the ZIP (zinc-iron permeases) family, which has a broad affinity for divalent metals with a preference for zinc (Grass et al., 2005). On the contrary, high-affinity zinc uptake systems, commonly mediated by members of the ATP-binding cassette (ABC) transporter superfamily, operate as a response to zinc shortage conditions. The expression levels of these transporters are controlled at the transcriptional level by Zur (zinc uptake regulator), a member of the ferric uptake transcriptional regulator (Fur) family. When enough Zn is present in the cell, Zn atoms bind to Zur protein, thus turning it into a repressor of genes involved in Zn uptake (Hantke, 2005; Mikhaylina et al., 2018; Kandari et al., 2021).

ABC transporter systems rely on three components: (i) a membrane-spanning permease, (ii) a cytosolic ATPase, and (iii) a periplasmic substrate-binding protein (SBP) that confers high affinity and selectivity to the system and forms a complex with the membrane component to deliver the metal into the cytosol (Cerasi et al., 2013; Blindauer, 2015; Bui and Inaba, 2024). The SBP structures are characterized by two independent globular α/β domains that interact to create a metal-binding pocket. Different kinds of SBPs have been described, and those from cluster A, distinguished by an α-helix that connects the two domains, comprise the subcluster A-I, including SBPs that directly interact with metal ions (Fe^2+^, Zn^2+,^ or Mn^2+^) (Berntsson et al., 2010). Structures of Zn^2+^-specific SBPs have revealed the existence of three conserved histidine residues that participate in metal coordination together with a conserved glutamate/aspartate residue or a water molecule (Banerjee et al., 2003; Yatsunyk et al., 2008; Ilari et al., 2011; Handali et al., 2015; Maunders et al., 2024). A glutamate residue at the same position of the third histidine participating in the coordination environment has been associated with Mn^2+^- or Fe^2+^-specific SBPs (Lawrence et al., 1998; Rukhman et al., 2005; Sun et al., 2009). Furthermore, some Zn^2+^-binding SBPs contain a histidine-rich domain that has been related to sensing/regulation or zinc acquisition functions (Banerjee et al., 2003; Wei et al., 2007; Neupane et al., 2017).

The symbiotic rhizobium-legume interaction, a key sustainable source of nitrogen in agro-ecosystems, requires adjusting the behavior of both partners by sophisticated plant- and bacterial-dependent mechanisms that lead to the formation of the root nodules (Ledermann et al., 2021; Hawkins and Oresnik, 2022). In the nodules, bacteria are surrounded by a plant-derived membrane, establishing intracellular symbiotic organelles (symbiosomes) where they differentiate into bacteroids, the symbiotic form of rhizobia that express the nitrogenase enzyme catalyzing the conversion of N_2_ into ammonia (Poole et al., 2018). Bacteroids are fully dependent on plant cells for the acquisition of nutrients, and establishment of the symbiosis triggers metal uptake mechanisms in the bacteroid, as many enzymes required for symbiotic performance are highly expressed metalloenzymes, including the nitrogenase, superoxide dismutases, and others required for bacteroid functions (Abreu et al., 2019; González-Guerrero et al., 2023). Furthermore, metals are transported to the bacteroid in complexes with organic acids, and availability of metals is sometimes compromised due to the speciation of the corresponding cation with strong plant-derived chelators (Moreau et al., 1995; Cacho et al., 2010). Although nodules constitute approximately 5% of the total plant biomass, they may contain 25%–30% of the total transition metals from the plant (González-Guerrero et al., 2023). Consequently, metal-related deficiencies, and in particular zinc soil deficiencies or alterations in the metal delivery pathways to the cytosol of plant-infected cells, have been shown to affect nodulation, symbiotic nitrogen fixation, and legume productivity (O’Hara, 2001; Abreu et al., 2017; León-Mediavilla et al., 2018).

In Gram-negative bacteria, zinc uptake is commonly mediated by the conserved high-affinity ZnuABC transporter system belonging to the ABC transporter superfamily (Patzer and Hantke, 2000), which plays an important role in pathogenic bacteria adaptation to zinc-limiting conditions. This system promotes survival and virulence in the host, in which metal availability is actively reduced in response to bacterial infection (Patzer and Hantke, 1998; Chen and Morse, 2001; Ammendola et al., 2007; Vahling-Armstrong et al., 2012; Akhtar and Turner, 2022). However, metal transport has been shown to involve more than a single system, and redundant functions for zinc uptake under these conditions have been observed (Desrosiers et al., 2010; Corbett et al., 2012; Cerasi et al., 2013; Plumptre et al., 2014; Pederick et al., 2015; Maunders et al., 2024). In contrast to the extensive studies in pathogenic bacteria, functional studies of zinc homeostasis in plant-associated bacteria are scarce. The requirement of *Sinorhizobium meliloti* ZnuABC for growth under zinc-limiting conditions has been demonstrated (Vahling-Armstrong et al., 2012), but its functional role during symbiosis has not been studied. In *Agrobacterium tumefaciens*, two zinc ABC transporters have been described (Bhubhanil et al., 2014; Chaoprasid et al., 2016), and the functional role of ZnuABC was revealed in the absence of the second transporter system, TroCBA, that predominantly plays zinc uptake functions under zinc-limiting conditions (Chaoprasid et al., 2016). In this system, the virulence of mutants affected by single mutations in the permease *troC* or in *znuA* was not affected (Bhubhanil et al., 2014; Chaoprasid et al., 2016), and the cooperation of both transporter systems in plant virulence has not been demonstrated. A transcriptome-based analysis revealed that the *Sinorhizobium fredii znuABC* operon was highly induced in soybean bacteroids as compared to free-living cells, and host-dependent upregulation patterns were also observed (Jiao et al., 2016; Jiao et al., 2018). The functional role of the SBP ZnuA has been shown to be dependent on the legume host in the *S. fredii*-soybean system (Jiao et al., 2018; Zhang et al., 2020), where accessory zinc transporters, such as ZIP transporters, contribute to rhizobia adaptation to the different hosts (Zhang et al., 2020).

*Rhizobium leguminosarum* bv. viciae (Rlv) UPM791 effectively nodulates legume genera belonging to the *Viciae* tribe (*Pisum*, *Vicia*, *Lathyrus*, and *Lens*) (Laguerre et al., 2003), but not all combinations are equally effective, and differences in symbiotic performance associated with the legume host have been described (Den Herder and Parniske, 2009). A proteomic analysis in bacteroids induced by Rlv UPM791 in pea and lentil nodules revealed the existence of a relevant number of proteins differentially expressed (Durán et al., 2021), suggesting that each legume host provides a different environment, inducing specific adaptive responses in the bacteria. Among these proteins, a potential metal-binding protein (RLV_3444, renamed as ZniA), a component of an ABC-transporter system (RLV_3442-3444), here designated as ZniCBA, was shown to be expressed at higher levels in pea bacteroids, suggesting that provision of this element to the bacteroid is more restrictive in the rhizobium-pea symbiosis. In this work, the functional role and expression analysis of this pea-specific SBP have been analyzed. We have demonstrated that ZniCBA is a zinc uptake transporter system that may replace the functional role of the ABC-transporter system ZnuABC under zinc-limiting conditions in free-living cells. The role of both transporter systems in the optimization of the symbiosis of Rlv with pea and lentil plants has also been established. The expression of the *zniCBA* operon and *znuA* gene has been shown to be regulated by zinc in a Zur-dependent manner. Both transporters were significantly induced in pea nodules, and their expression pattern suggests that *zniCBA* might function as an auxiliary zinc uptake system under zinc starvation conditions and might play a relevant role in the adaptation of Rlv to its legume host.

## Materials and methods

### Bacterial strains, plasmids, and growth conditions

Strains and plasmids used in this study are listed in **Supplementary Table S1**. *R. leguminosarum* strains were routinely grown at 28°C in yeast mannitol broth (YMB) (Vincent, 1970), tryptone-yeast extract (TY) (Beringer, 1974), *Rhizobium* minimal media (Rmin) (O’Gara and Shanmugam, 1976), or universal minimal salt (UMS) medium (Wheatley et al., 2017). *Escherichia coli* strains were grown at 37°C in LB medium (Bertani, 1951). *E. coli* DH5α was used for standard cloning procedures (Hanahan, 1983), and *E. coli* S17.1 (Simon et al., 1983) for conjugative plasmid transfer between *E. coli* and *R. leguminosarum*. Antibiotic concentrations used were as follows (µg ml^−1^): ampicillin, 100; kanamycin, 50; tetracycline, 5 (for *R. leguminosarum*) or 10 (for *E*. *coli*).

For growth curve analyses, bacterial cultures were grown in zinc-free UMS medium up to an OD_600_ of 0.6 and diluted to an initial OD_600_ of 0.01 in fresh zinc-free UMS or the same medium containing 50 µM EDTA with or without 50 µM ZnSO_4,_ MnSO_4,_ or FeSO_4_ supplementation. Growth curves were determined with cell cultures incubated in 100-well Honeycomb plates (Growth Curves Ltd., Piscataway, NJ, USA) with continuous double-orbital shaking in a Bioscreen C Pro device (Growth Curves Ltd., Piscataway, NJ, USA) at 28°C, with OD_600_ measurement intervals every 30 min for 30 h.

### Mutants and plasmid construction

DNA purification, digestion with restriction enzymes, ligation, gel electrophoresis, amplification by polymerase chain reaction (PCR), and *E. coli* transformation were performed as described in standard protocols (Sambrook and Russell, 2001). Primers used for mutants and plasmid construction are listed in **Supplementary Table S2**.

Derivatives of Rlv SPF25 incorporating *zniA* and *zniCBA* mutations were generated by homologous recombination using the suicide vector pK18*mobsac* (Schäfer et al., 1994). For this purpose, a first round of PCR reactions amplified two DNA fragments of *ca.* 1 Kb corresponding to up- and downstream regions of the *zniA* (*rlv_3444*) gene using primers P1_rlv_3444_BamHI/P2_rlv_3444 and P3_rlv_3444/P4_rlv_3444_HindIII, respectively, and genomic DNA from the Rlv UPM791 strain as a template. The two PCR products of the flanking DNA regions of the gene were linked via an overlapping GC-rich (C_5_G_5_C_5_) sequence, previously incorporated using P2_rlv_3444 and P3_rlv_3444 primers, by a fusion PCR (Cha-aim et al., 2009) using primers P1_rlv_3444_BamHI/P4_rlv_3444_HindIII. Following the same procedure, regions covering *zniC* (*rlv_3442*) upstream and *zniA* (*rlv_3444*) downstream were amplified using primers P1_rlv_3442-44_BamHI/P2_rlv_3442–44 and P3_rlv_3444/P4_rlv_3444_HindIII, respectively. A fusion PCR with primers P1_rlv_3442-44_BamHI/P4_rlv_3444_HindIII was performed to amplify the fragment carrying the *zniCBA* deletion. Fusion PCR products containing *zniA* and *zniCBA* deletions were cloned separately in pBlueScript-II KS+ as BamHI-HindIII restriction fragments, sequenced, and moved to the pK18*mobsac* suicide vector. The resulting plasmids, pK18.ZniA and pK18.ZniCBA, were introduced independently into the SPF25 strain by conjugation, and homologous recombination by a double crossover event was selected by the *sacB* system in *Rhizobium* minimal medium (Schäfer et al., 1994). Gene deletions were verified by PCR and sequencing analysis, and the transconjugant strains carrying the mutations in *zniA* and *zniCBA* were designated as UPM1629 and UPM1631, respectively.

To generate an in-frame deletion in the *znuA* gene, regions up- and downstream of the *znuA* gene were PCR amplified using primers P1_ZnuA_BamHI/P2_ZnuA and P3_ZnuA/P4_ZnuA_HindIII, respectively, and UPM791 genomic DNA as a template. The two PCRs were linked via an overlapping GC-rich sequence by a fusion PCR using primers P1_ZnuA_BamHI/P4_ZnuA_HindIII to amplify the fragment containing the deletion of the *znuA* gene. The resulting DNA fragment was digested with BamHI-HindIII restriction enzymes and cloned in pBlueScript-II KS+, sequenced, and then moved to the pK18*mobsac* suicide vector. The new plasmid generated (pK18.ZnuA) was introduced into SPF25 strain and into derivative strains UPM1629 (Δ*zniA*) and UPM1631 (Δ*zniCBA*) by conjugation, and homologous recombinations were selected by the *sacB* system. Gene deletion was verified by PCR and sequencing analysis, and the transconjugant strains carrying mutations in *znuA*, *znuA/zniA*, and *znuA/zniCBA* were designated as UPM1628, UPM1630, and UPM1632, respectively.

To generate mutant derivatives bearing a deletion in the *zur* gene, two DNA regions up- and downstream of the gene were amplified by PCR using primer pairs P1_Zur/P2_Zur and P3_Zur/P4_Zur, respectively. The resulting DNA fragments were fused by an overlapping GC sequence by PCR using P1_Zur/P4_Zur primers. The PCR product carrying the *zur* deletion was cloned in plasmid pCR2.1TOPO, sequenced, and moved to the pK18*mobsac* vector as an XbaI-BamHI restriction fragment, rendering the pK18.Zur plasmid. This construction was introduced into SPF25 and into its derivative strain UPM1630 (Δ*znuA*Δ*zniA*), and homologous recombination was selected by the *sacB* system, giving rise to UPM1633 (Δ*zur*) and UPM1634 (Δ*zur*Δ*znuA*Δ*zniA*). Gene deletion was checked by PCR and sequencing analysis.

For analysis of promoter expression, transcriptional gene fusions were generated with the promoterless *gusA* gene in plasmid pLMB51 (Tett et al., 2012). DNA fragments containing the upstream regions of *zniC* and *znuA* genes were PCR amplified with primers Prom1_rlv_3442-44_BamHI_F/Prom5_rlv_3442-44_XbaI_R and Prom_ZnuA_BamHI_F/Prom_ZnuA_XbaI_R, respectively, and UPM791 genomic DNA as a template. PCR products were cloned separately into pBlueScript-II KS+ plasmid using BamHI-XbaI restriction sites, sequenced, and moved to pLMB51 plasmid, thus generating pLMBZniCBA and pLMBZnuA plasmids, respectively (Tett et al., 2012). For regulatory analysis of the expression of the *zniCBA* operon, truncated forms of the DNA region upstream of the operon containing 537, 258, and 88 bp from the ATG codon were amplified with primers Prom2_rlv_3442-44_BamHI_F/Prom5_rlv_3442-44_XbaI_R, Prom3_rlv_3442-44_BamHI_F/Prom5_rlv_3442-44_XbaI_R, and Prom4_rlv_3442-44_BamHI_F/Prom5_rlv_3442-44_XbaI_R, respectively. PCR products were cloned separately into pBlueScript-II KS+ plasmid, sequenced, and moved to pLMB51 plasmid as a BamHI-XbaI restriction fragment, rendering pLMB537.ZniCBA, pLMB258.ZniCBA, and pLMB88.ZniCBA, respectively.

For the construction of pBBRZniA, the DNA region upstream of the *zniC* gene and the *zniA* gene from its 16 bp upstream sequence were amplified with primers Prom3_rlv_3442-44_BamHI_F/P2_Com_rlv_3444_R and P3_Com_rlv_3444_F/P4_Com_rlv_3444_XbaI_R, respectively, and UPM791 genomic DNA as a template. The two PCRs were linked by an overlapping GC-rich sequence through a fusion PCR using primers Prom3_rlv_3442-44_BamHI_F/P4_Com_rlv_3444_R. The same procedure was performed for the construction of pBBRZniA_ST_, but the P4_Com_rlv_3444Strep_XbaI_R, including the sequence coding for StrepTag II peptide (WSHPQFEK) for in-frame fusion of the tag sequence to the 3´end of the gene, was used. To generate the pBBRZnuA plasmid, DNA covering the *znuA* gene and its promoter region was PCR-amplified with Prom_ZnuA_BamHI_F/Com_ZnuA_XbaI_R primers. The PCR products obtained were cloned separately in the pBlueScript-II KS+ vector as BamHI-XbaI restriction fragments, sequenced, and moved to the broad host range vector pBBR1MCS-2 (Kovach et al., 1995). Site-directed mutagenesis of the *zniA_ST_* gene was performed in pBBRZniA_ST_ using complementary oligonucleotides with adequate centered nucleotide substitutions (**Supplementary Table S2**) and DpnI digestion to eliminate the native template. Substitution of target nucleotides as well as the absence of additional mutations was verified by sequencing. The pLMBcZni plasmid was generated by amplifying by PCR the *zniCBA* operon and its promoter region using primers Prom1_rlv_3442-44_BamHI_F and rlv_3442-44_XbaI_R, and UMP791 genomic DNA as template. The PCR product was cloned in the pBluescript-II KS+ plasmid, sequenced, and then moved as a BamHI-XbaI fragment to the pLMB51 plasmid.

### Plant assays

Pea (*Pisum sativum* L. cv. Frisson) and lentil (*Lens culinaris* cv. Magda) were surface-sterilized, pregerminated, planted in Leonard jar assemblies under bacteriologically controlled conditions, and inoculated with 1 ml of early stationary phase bacterial cultures grown in YMB as previously described (Sánchez-Cañizares et al., 2011). Plants were grown in a greenhouse with a standard nitrogen-free plant nutrient solution (Brito et al., 1994). When required, the nutrient solution was supplemented with ZnSO_4_ at the desired concentration. Plants were grown at 18/25°C night/day with 16/8 h of light/dark photoperiod and harvested 21 days (pea) or 28 days (lentil) after inoculation. Shoots were collected and dried (60°C for 48 h), and shoot dry weight was determined. Shoot total nitrogen content was measured using a TruMac C/N analyzer (Leco Corporation).

### β-Glucuronidase activity assays

β-Glucuronidase activity assays were performed with free-living cells and bacteroids as described in Lodwig et al. (2004) using X-GlcA (5-bromo-4-chloro-3-indolyl-β-D-glucuronide) as substrate. Values of activity were calculated as OD_420_ min^−1^ (mg protein)^−1^ and expressed as Miller units. Bacteroids were obtained from the nodules as previously described (Cacho et al., 2010). Protein content was obtained following the bicinchoninic acid method (Smith et al., 1985) after alkaline digestion of cells at 90°C in 2 N NaOH for 10 min using bovine serum albumin as the standard.

### RNA extraction and qRT-PCR

RNA was extracted from nodule samples as described in Ballesteros-Gutiérrez et al. (2023). RNA concentration was quantified with a NanoDrop spectrophotometer and tested for possible DNA contamination by PCR using the RNA samples as templates and primers specific for *rpoD* (rpoD_qPCR_F/rpoD_qPCR_R). RNA integrity was confirmed by electrophoresis in a 1% agarose gel. cDNA was synthesized from 500 ng of DNA-free RNA using the PrimeScript RT reagent kit (Takara, Saint-Germain-en-Laye, France), supplemented with RNase out (Invitrogen, Thermo Fisher Scientific, Massachusetts, USA) following the manufacturer’s specifications. Quantitative real-time reverse transcription polymerase chain reaction (qRT-PCR) was performed with Power SyBR Green master mix (Applied Biosystems) and primers rlv_3444_qPCR_F/rlv_3444_qPCR_R for *zniA*, znuA_qPCR_F/znuA_qPCR_R for *znuA*, hupL_qPCR_F/hupL_qPCR_R for *hupL*, and rpoD_qPCR_F/rpoD_qPCR_R for *rpoD* genes. Gene expression levels were normalized to the *rpoD* reference gene (Ballesteros-Gutiérrez et al., 2023). Determinations were performed with RNA extracted from three independent biological samples, with the threshold cycle (*C*_T_) determined in triplicate. The relative levels of transcription were calculated using the 2^–ΔΔ^ *^C^* ^T^ method (Livak and Schmittgen, 2001).

### Bioinformatic analysis

Protein sequences were obtained from the GenBank database. Multiple sequence alignment was created with SnapGene 6.2 software using the Clustal Omega multiple alignment algorithm v.1.2.4 (Chenna et al., 2003). The phylogenetic tree of the protein alignment was analyzed with MEGA 12 (Tamura et al., 2021) using a neighbor-joining algorithm method (Saitou and Nei, 1987) with bootstrapping (1,000 iterations). The Uniprot database (https://www.uniprot.org/) was used to perform protein BLAST and amino acid sequence identity analysis. Zur box prediction from DNA upstream of *zniCBA* and *znuA* genes were generated based on the conserved sequence motifs using the bioinformatics tool Multiple EM for Motif Elicitation (MEME) (Bailey et al., 2006). The analysis included the 42 Zur-binding motifs from *Rhizobiales* obtained from the Regprecise database of the curated inferences of regulons in prokaryotic genomes (Novichkov et al., 2009). Structural prediction of the mature RLV_3444 protein (25–299 residues) was carried out using the AlphaFold 3 server (Abramson et al., 2024). Structural comparison with the PDB database was performed at the DALI server (http://ekhidna2.biocenter.helsinki.fi/dali/) (Holm et al., 2023). The program was run with the default server parameters. Alignments for further data analysis and figure preparation were done in Pymol (v.3.1.3.1, Schrödinger). Published protein structures were obtained from the PDB database.

### Statistical analysis

Statistical analysis was performed by analysis of variance (ANOVA) linear model test following completely random design. Multiple comparisons of means were analyzed as indicated in the corresponding legend figure. Statistical analysis and graph generation were performed with GraphPad Prism 8 (GraphPad Software).

## Results

### Identification of two putative zinc ABC transporter systems in *R*. *leguminosarum* bv. viciae UPM791 genome

**Figure 1:**
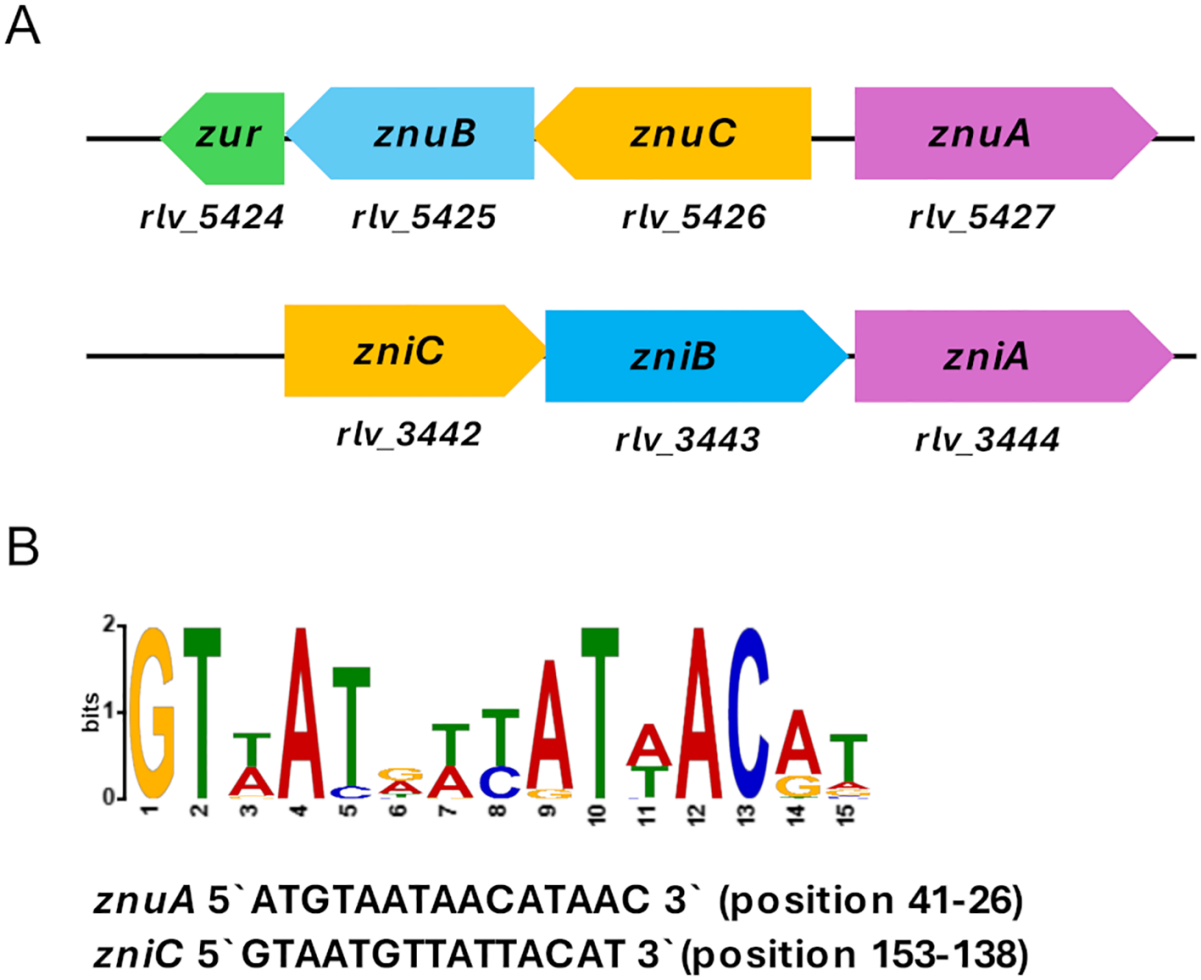
Genomic context of *znuA* and *zniA* genes in *R. leguminosarum* bv. viciae UPM791. **(A)** Gene designations of putative *znuABC* and *zniCBA* operons, and *zur* gene in Rlv UPM791 genome are shown below the genetic map. **(B)** The Zur consensus motif determined by comparison of promoter DNA from *Rhizobiales* and Rlv UPM791 *znuA* and *zniC* genes using MEME. More prominent letters denote more frequent usage in the motif. The sequences of Zur boxes and the position indicated as bp upstream of the translational start sites in the promoter regions of *znuA* and *zniC* genes are indicated.

Inspection of the Rlv UPM791 genome reveals the presence of a chromosomic operon (*rlv_5427-5425*) encoding the putative high-affinity zinc transporter system ZnuABC. The *znuA* (*rlv_5427*) gene is oriented in the opposite direction to the *znuC* (*rlv_5426*) and *znuB* (*rlv_5425*) genes (**Figure 1A**). Rlv ZnuA shares a high amino acid sequence identity with its homologues in *S*. *fredii*, *A*. *tumefaciens* and *S*. *meliloti* (identities of 69.1%, 67.31%, and 66.56%, respectively) (**Supplementary Figure S1**). A Zur-like transcriptional regulator is encoded by gene *rlv_ 5424* located immediately downstream of the *znuB* gene (**Figure 1A**). RLV_5424 is very similar to Zur from *A. tumefaciens* C58, *S*. *fredii* CCBAU 45436 and *S. meliloti* 1021 strains (74.24%, 72.52%, and 70.23% sequence identities, respectively). A Zur box containing a 15 bp sequence was identified upstream *znuA* gene, very similar to the conserved Zur motifs of *Rhizobiales*, localized 41 bp upstream of the translational start site of the gene (**Figure 1B**).

The Rlv UPM791 genome encodes a metal ABC transporter system containing the host-dependent metal-binding protein RLV_3444 (Durán et al., 2021). This protein is encoded by the chromosomic *rlv_3444* gene, which is part of an operon with a typical organization of bacterial ABC permeases and also encodes an ATP-binding protein (*rlv_3442*) and a permease (*rlv_3443*) (**Figure 1A**). In addition, a conserved Zur motif was also identified 153 bp upstream of the *rlv_3442* gene. A comparison of the RLV_3444 amino acid sequence with protein databases revealed a highly significant level of conservation (over 75% identity) with proteins annotated as metal, Zn, or Zn/Mn ABC transporter SBPs in the genomes of several members of *Rhizobiaceae*. Furthermore, RLV_3444 exhibits a high amino acid sequence identity with the functionally characterized SBPs from *K. pneumoniae* ZniA (53.42% sequence identity)*, A. tumefaciens* TroA (41.61% identity) and *Paracoccus denitrificans* AztC (40.88% identity), previously shown to participate in zinc uptake (Chaoprasid et al., 2016; Handali et al., 2015; Maunders et al., 2024) (**Supplementary Figure S1**). Sequence alignments revealed that RLV_3444 contains the three fully conserved histidine residues (H62, H127, and H193, RLV_3444 numbering) required for the coordination of a zinc atom in the crystal structures of *K*. *pneumoniae* ZniA and *P*. *denitrificans* AztC (**Supplementary Figure S2**). In RLV_3444, a glutamate at position 268, conserved in *K. pneumoniae* ZniA, where it participates in zinc coordination (Maunders et al., 2024), was also identified. In contrast, the short central histidine-rich domain present in *P*. *denitrificans* AztC and *A. tumefaciens* TroA and associated to Zn^2+^ specificity (Blindauer, 2015) is not present either in RLV_3444 or in *K*. *pneumoniae* ZniA.

**Figure 2:**
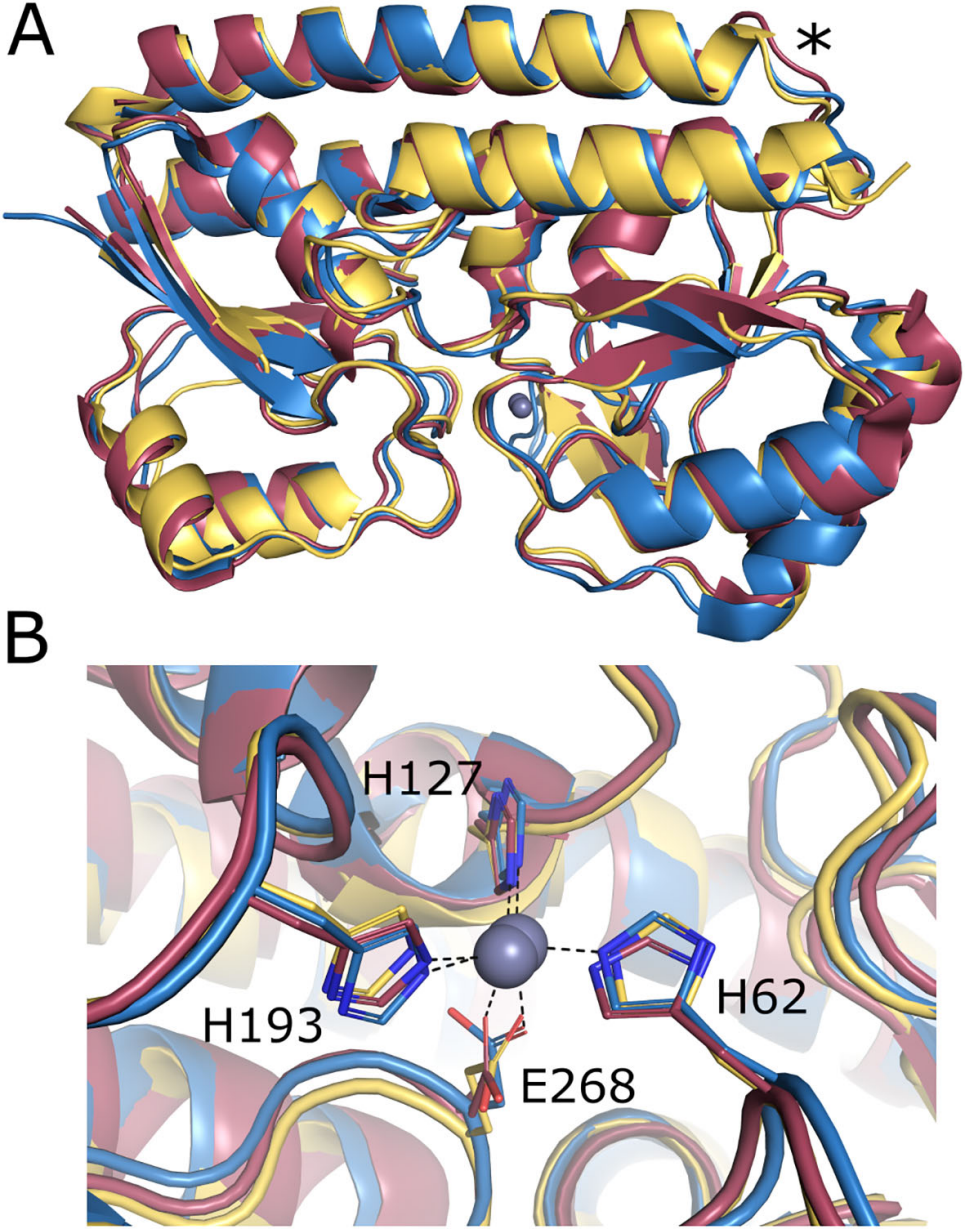
*In-silico* modelled structure of *R. leguminosarum* ZniA. **(A)** Superposition of the Alphafold model structure of *R*. *leguminosarum* ZniA (yellow, residues 25-299) and the crystal structure of *K. pneumoniae* ZniA (red, PDB: 8SVC) and *P. denitrificans* AztC (blue, PDB: 5W57). The cluster A rigid α-helix is marked with an asterisk. **(B)** Detail for metal binding site of *R. leguminosarum* ZniA (yellow) aligned with *K. pneumoniae* ZniA (red, PDB: 8SVC) and *P. denitrificans* AztC (blue, PDB: 5W57). The zinc atom is shown in grey.

Structural prediction of mature RLV_3444 protein (25–299 residues) was carried out using the AlphaFold 3 server, and the model obtained was compared to the available structures in the PDB database using the Dali server. Proteins showing the best structural homologies with RLV_3444 were zinc-, manganese-, and iron-binding proteins, with the highest scores for *K. pneumoniae* ZniA (PDB id: 8SVC; Maunders et al., 2024) and *P. denitrificans* AztC (PDB id: 5W57; Neupane et al., 2017) (**Supplementary Table S3**), suggesting similar functions for these proteins. Structural alignment of RLV_3444 with AztC and ZniA has revealed that it shares the two independent domains that interact to create a metal-binding pocket between them connected by a rigid α-helix characteristic of the cluster A SBPs-associated family (Berntsson et al., 2010) (**Figure 2A**). Structural alignments have also shown a highly conserved metal binding site (**Figure 2B**).

Taken together, these results suggest that RLV_3444 is a potential zinc-binding protein of an ABC transporter system. Based on the bioinformatic analysis and the functional and regulatory role of this protein (see below), the corresponding gene was designated as *zniA* (zinc import), and we also designated the *rlv_3442–3444* operon as *zniCBA* (**Figure 1**).

### ZniCBA replaces the functional role of ZnuA in *R*. *leguminosarum* free-living cells under zinc-limiting conditions

**Figure 3:**
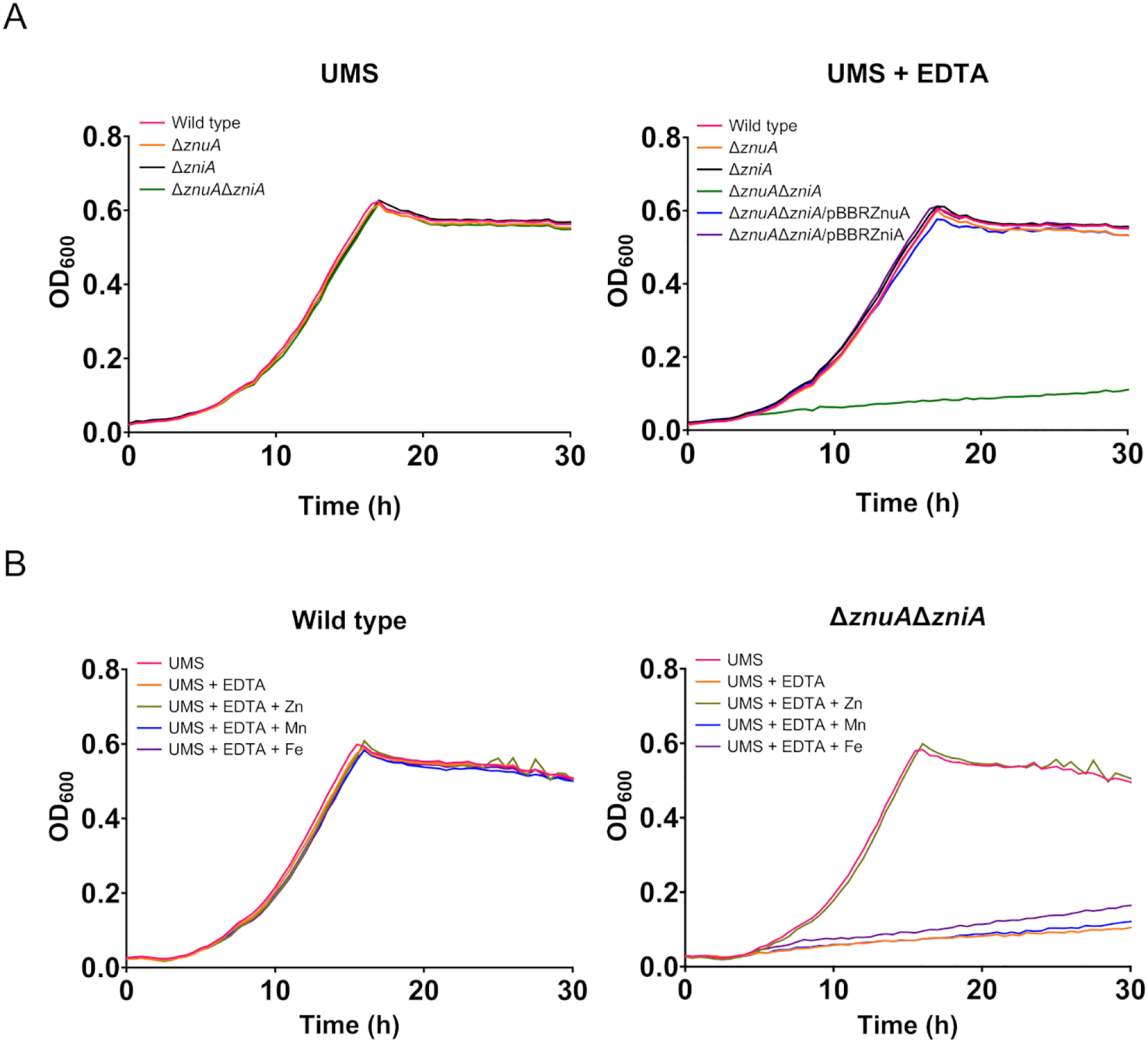
Growth curves of Rlv strains under metal-limiting conditions. **(A)** Effect of ZnuA and ZniA on Rlv SPF25 growth. Rlv strains were grown until the stationary phase in zinc-depleted (no zinc added) UMS medium (UMS) or in the same medium supplemented with EDTA (50 µM EDTA; UMS+EDTA). **(B)** Effect of metal supplementation on the growth of Rlv strains. Rlv strains were grown until the stationary phase in zinc-depleted (no zinc added) UMS medium (UMS), in EDTA-chelated medium (no zinc added, 50 µM EDTA; UMS+EDTA) or in the same medium supplemented with 50 µM of either ZnSO_4_, MnSO_4_ or FeSO_4_, as indicated. Each OD determination represents the mean of three replicates. All standard errors were below 10%. Strains: wild type (SPF25, pBBR), Δ*znuA* (UPM1628, pBBR), Δ*zniA* (UPM1629, pBBR), Δ*znuA*Δ*zniA* (UPM1630, pBBR). *znuA* and *zniA* were provided cloned in pBBR1MCS-2 as indicated (pBBRZnuA and pBBRZniA plasmids, respectively). pBBR: pBBR1MCS-2.

In order to investigate the functional role of ZniA under free-living conditions, growth curves of SPF25 and its derivatives UPM1628 (Δ*znuA*), UPM1629 (Δ*zniA*), and UPM1630 (Δ*znuA*Δ*zniA*) strains were determined in UMS medium with no zinc added. The results indicated that all bacterial strains exhibited similar growth under these zinc-depleted conditions (**Figure 3A**). Atomic absorption spectromety analysis of zinc in the “Zn-free” medium revealed the presence of *ca.* 1 µM Zn^2+^ traces, likely too high to induce real zinc deprivation conditions (Outten and O’Halloran, 2001), so we added EDTA (50 μM) to chelate zinc cations. In the presence of EDTA, the growth of *znuA*- and *zniA*-deleted single mutants was similar to that observed in the wild-type strain. Conversely, the bacterial strain carrying deletions in both *znuA* and *zniA* genes (UPM1630) showed a clearly defective growth phenotype (**Figure 3A**). This phenotype was fully reverted by the expression of *znuA* or *zniA* genes in pBBRZnuA or pBBRZniA plasmids, respectively. Furthermore, the phenotype of the double mutant was also reverted by supplementing the medium with Zn^2+^ (50 µM ZnSO_4_) indicating that the defective phenotype of UPM1630 was due to its inability to take up zinc (**Figure 3B**). Supplementation of EDTA-containing medium with other metals (Mn^2+^ and Fe^2+^) did not restore the growth of the *znuA*/*zniA*-deficient derivative UPM1630 (**Figure 3B**), indicating that the growth defect was specifically related to a zinc deficiency. Altogether, these data indicate that ZniA plays a functional role similar to that of ZnuA, both proteins being able to support wild-type levels of growth under zinc-limiting conditions in *R. leguminosarum* free-living cells.

### ZniA and ZnuA are not interchangeable for providing zinc to Znu permease

**Figure 4:**
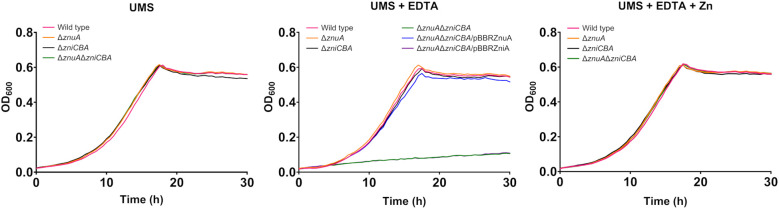
Effect of ZniA expression on the complementation of ZnuA under metal limiting conditions. Rlv strains were grown until the stationary phase in zinc-depleted (no zinc added) UMS medium (UMS), in EDTA-chelated medium (no zinc added, 50 µM EDTA; UMS+EDTA) or in the same medium supplemented with 50 µM ZnSO_4_ (UMS+EDTA+Zn). Each OD determination represents the mean of three replicates. All standard errors were below 10%. Strains: wild type (SPF25, pBBR), Δ*znuA* (UPM1628, pBBR), Δ*zniCBA* (UPM1631, pBBR), Δ*znuA*Δ*zniCBA* (UPM1632, pBBR). *znuA* and *zniA* were provided cloned in pBBR1MCS-2 as indicated (pBBRZnuA and pBBRZniA plasmids, respectively). pBBR: pBBR1MCS-2.

With the aim to consider the possibility of cross-talk between Zni and Znu systems *via* their respective SBPs, we constructed a mutant (UPM1631) carrying a deletion of the whole *zniCBA* operon. This mutant showed normal growth under all conditions tested, as expected from the presence of an active Znu system (**Figure 4**). These results indicate that ZniCBA and ZnuABC were functionally redundant under metal-limitation conditions in the presence of EDTA (0 µM ZnSO_4_, 50 µM EDTA). In order to check whether ZniA could be providing Zn^2+^ to the bacterial cell *via* the Znu permease in the absence of ZnuA, a *znuA* deletion was generated in this *zniCBA* mutant, thus resulting in the UPM1632 strain. The growth of the UPM1632 double mutant was similar to that observed in the wild-type strain in UMS under zinc-depleted conditions (0 µM ZnSO_4_ added), but it was impaired in the presence of EDTA. The defective phenotype was reverted back to wild-type levels by supplementing the medium with ZnSO_4_ (50 µM) and also by the expression of the *znuA* gene from the pBBRZnuA plasmid. In contrast, such complementation was not induced by the expression of *zniA* from pBBRZniA. As a control, we checked that the UPM1632 defective growth phenotype under metal-chelated conditions was rescued to wild-type levels with the expression of ZniCBA under the control of its own promoter in the pLMBcZni plasmid (**Supplementary Figure S3** **).** These data demonstrate that ZnuA function cannot be substituted by ZniA when Zn provision depends on Znu permease.

### Functional analysis of ZniA residues potentially involved in metal coordination

**Figure 5:**
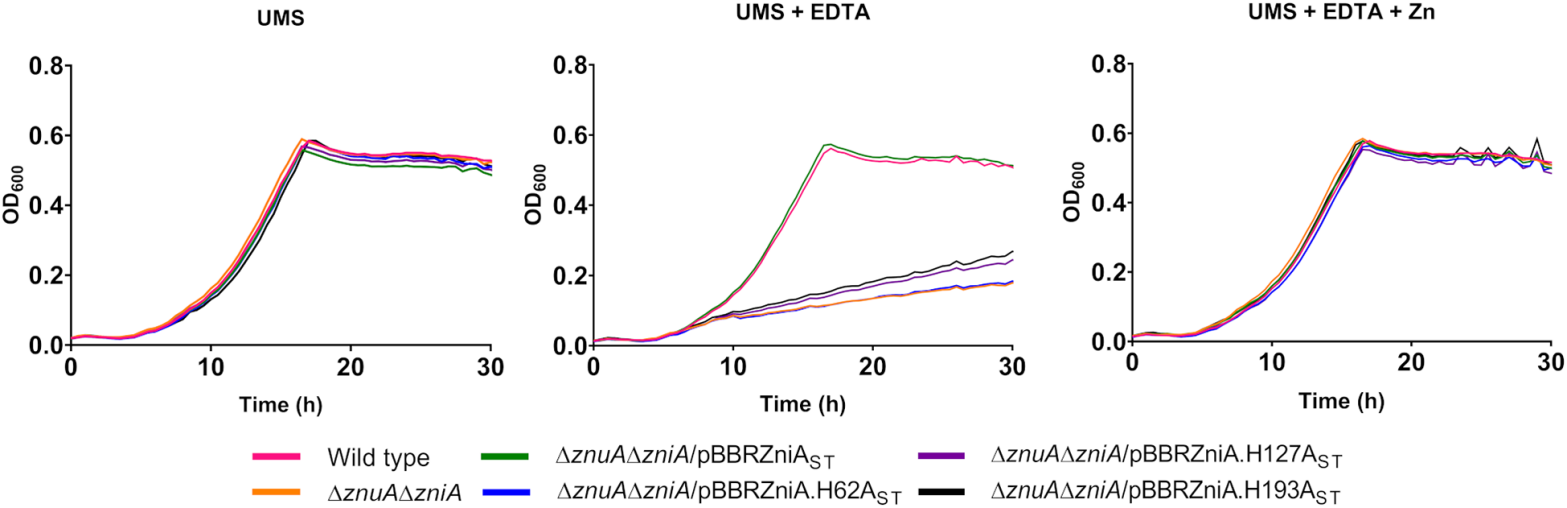
Functional analysis of conserved histidine residues from Rlv ZniA. The indicated Rlv strain complemented with plasmid-based *zniA* or with *zniA* versions with the indicated alterations in histidine residues were grown until stationary phase in zinc-depleted (no zinc added) UMS medium (UMS), in EDTA-chelated medium (no zinc added, 50 µM EDTA; UMS+EDTA) or in the same medium supplemented with 50 µM ZnSO_4_ (UMS+EDTA+Zn). Each OD determination represents the mean of three replicates. All standard errors were below 10%. Strains: wild type (SPF25, pBBR), Δ*znuA*Δ*zniA* (UPM1630, pBBR). pBBR: pBBR1MCS-2.

The potential involvement of ZniA histidine residues (H62, H127, and H193) in ZniA function has been investigated. To carry out these studies, plasmids expressing ZniA_ST,_ incorporating a C-terminal Strep-Tag affinity tail for future monitoring and purification purposes, and variants harboring H62A, H127A, and H193A changes were generated and transferred separately by conjugation into the *znuA/zniA*-deleted strain UPM1630. Bacterial cultures of the corresponding transconjugant strains were analyzed for growth in UMS medium under zinc-depleted (0 µM ZnSO_4_), metal-chelated (0 µM ZnSO_4_, 50 µM EDTA), and zinc-repleted (50 µM ZnSO_4_, 50 µM EDTA) conditions. The growth of all strains was similar to that observed in the SPF25 strain under zinc-depleted conditions (**Figure 5**). The expression of ZniA_ST_ in pBBRZniA_ST_ complemented the defective growth phenotype of the UPM1630 strain up to wild-type levels under zinc-chelated conditions, indicating that the incorporation of the affinity tail did not affect the function of the protein. In these experiments, we observed that *zniA_ST_* carrying the mutations H62A, H127A, or H193A did not complement the Δ*zniA*Δ*znuA* mutant for growth in the medium containing EDTA (0 µM ZnSO_4_, 50 µM EDTA). The defective growth associated with the mutation in the histidine residues was restored with the addition of zinc to the EDTA-chelated medium. These results suggest that these histidine residues are critical for ZniA function, likely by contributing to metal coordination along the transport process.

### Rlv ZniA and ZnuA contribute to symbiotic performance in pea and lentil plants

**Figure 6:**
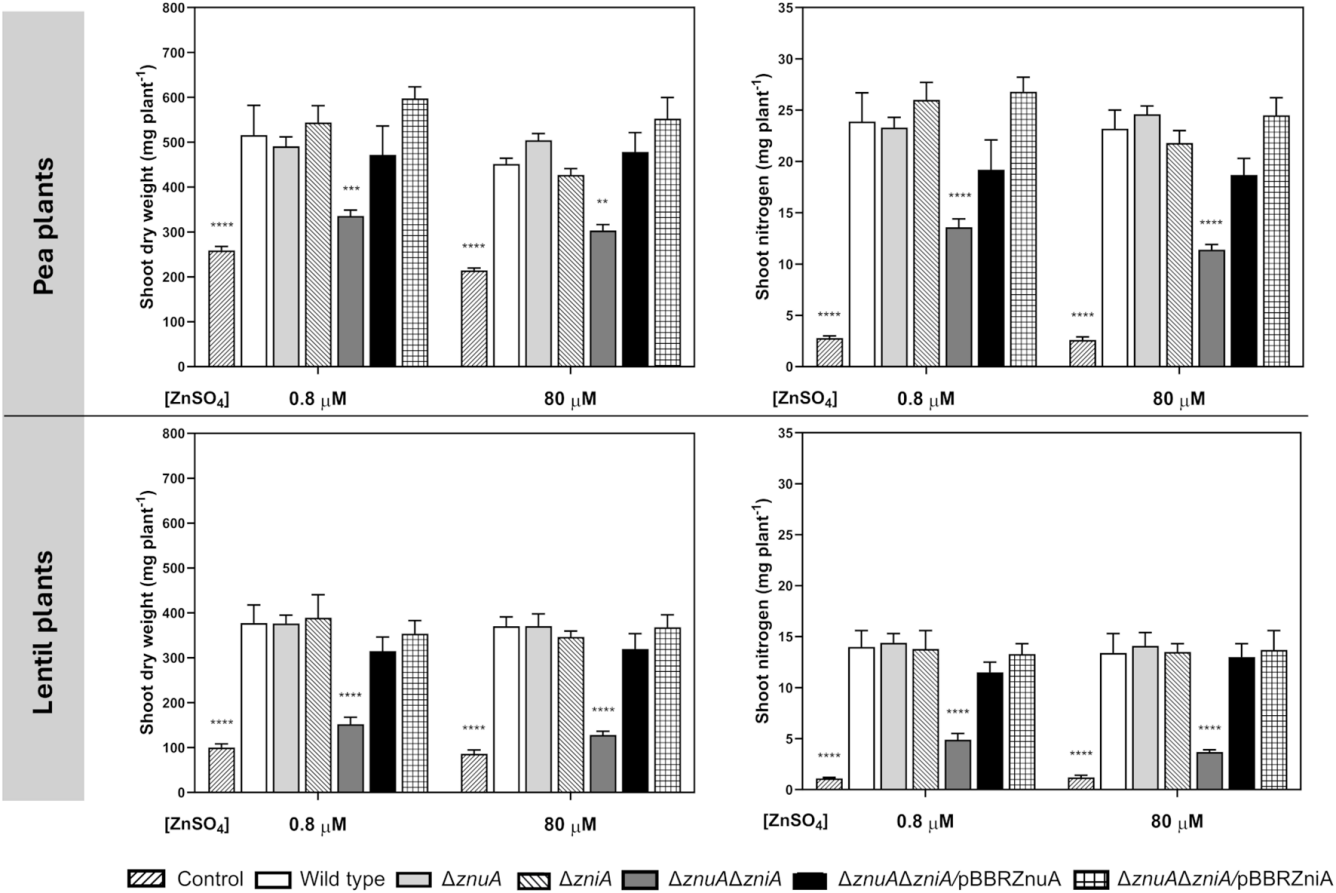
Effect of ZnuA and ZniA on the symbiotic performance of Rlv SPF25 with pea and lentil as host plants. Histograms represent the shoot dry weight and shoot nitrogen content of pea and lentil plants inoculated with the indicated Rlv strains. Plants were grown in a N-free standard nutrient solution (0.8 µM ZnSO_4_) or in the same solution containing 80 µM ZnSO_4_ as specified. Bars are the mean of four replicates ± standard error. Data were analyzed by one-way ANOVA and Dunnett´s test for multiple comparisons of means with wild type strain. **P < 0.01, ***P < 0.001, ****P < 0.0001. Control: uninoculated plants. Strains: wild type (SPF25), Δ*znuA* (UPM1628), Δ*zniA* (UPM1629), Δ*znuA*Δ*zniA* (UPM1630), Δ*znuA*Δ*zniA*/pBBRZnuA (UPM1630, pBBRZnuA), Δ*znuA*Δ*zniA*/pBBRZniA (UPM1630, pBBRZniA).

The metal-binding protein ZniA was shown to be overexpressed in pea bacteroids, with protein expression levels *ca*. twofold higher in pea than in lentil bacteroids (Durán et al., 2021). Our proteomic analysis also revealed that ZnuA was detected in bacteroids from pea but not from lentil (Durán et al., 2021). Furthermore, previous studies revealed a host-dependent contribution of ZnuA in *S. fredii-*soybean system (Jiao et al., 2018; Zhang et al., 2020). This prompted us to study the potential requirement for ZniA and ZnuA for efficient symbiosis of Rlv with pea and lentil plants. To carry out these studies, plants inoculated with Rlv SPF25 and its derivatives UPM1628 (Δ*znuA*), UPM1629 (Δ*zniA*), and UPM1630 (Δ*znuA*Δ*zniA*) strains were grown in a standard N-free nutrient solution (0.8 µM ZnSO_4_) or in the same solution containing 80 µM ZnSO_4_. Symbiotic performance of *znuA*- and *zniA*-deleted strains was similar to that of the wild-type strain in both legumes under either standard- or high-zinc concentrations (**Figure 6**). In contrast, pea and lentil plants inoculated with *znuA*/*zniA* double mutant UPM1630 accumulated significantly less shoot dry weight and less nitrogen in comparison to the wild-type strain at both zinc concentrations tested. This result indicates that high-affinity zinc transport is essential for optimal development of symbiosis of *R. leguminosarum* with legume plants. The impaired phenotype of the UPM1630 strain was complemented by the overexpression of wild-type copies of ZniA or ZnuA in pBBRZniA or pBBRZnuA plasmids, respectively (**Figure 6**). In contrast, the observed impairment of plant growth in the double mutant was not corrected by the addition of additional Zn^2+^ to the nutrient solution (80 μM ZnSO_4_, 100-fold higher than the original concentration). This observation indicates that, in the absence of functional Znu and Zni systems, potential alternative metal transporters do not provide the bacteroids with enough zinc for the symbiotic performance of this strain. These results indicate that both ZnuABC and ZniCBA transporter systems play interchangeable, essential functional roles in the provision of Zn^2+^ to the bacteroids under symbiotic conditions.

### Analysis of *zniCBA* and *znuA* genes expression under free-living conditions

In order to investigate the regulatory mechanisms governing the expression of *zni* and *znu* genes, independent transcriptional fusions to DNA upstream from the *zniCBA* operon and from the *znuA* gene to a promoterless *gusA* reporter gene in the pLMB51 plasmid were constructed, thus generating plasmids pLMBZniCBA (*zniCBA´-gusA*) and pLMBZnuA (*znuA*´-*gusA*). These plasmids were transferred separately by conjugation to SPF25 and to its derivatives UPM1628 (Δ*znuA*), UPM1629 (Δ*zniA*), and UPM1630 (Δ*znuA*Δ*zniA*) strains.

**Figure 7:**
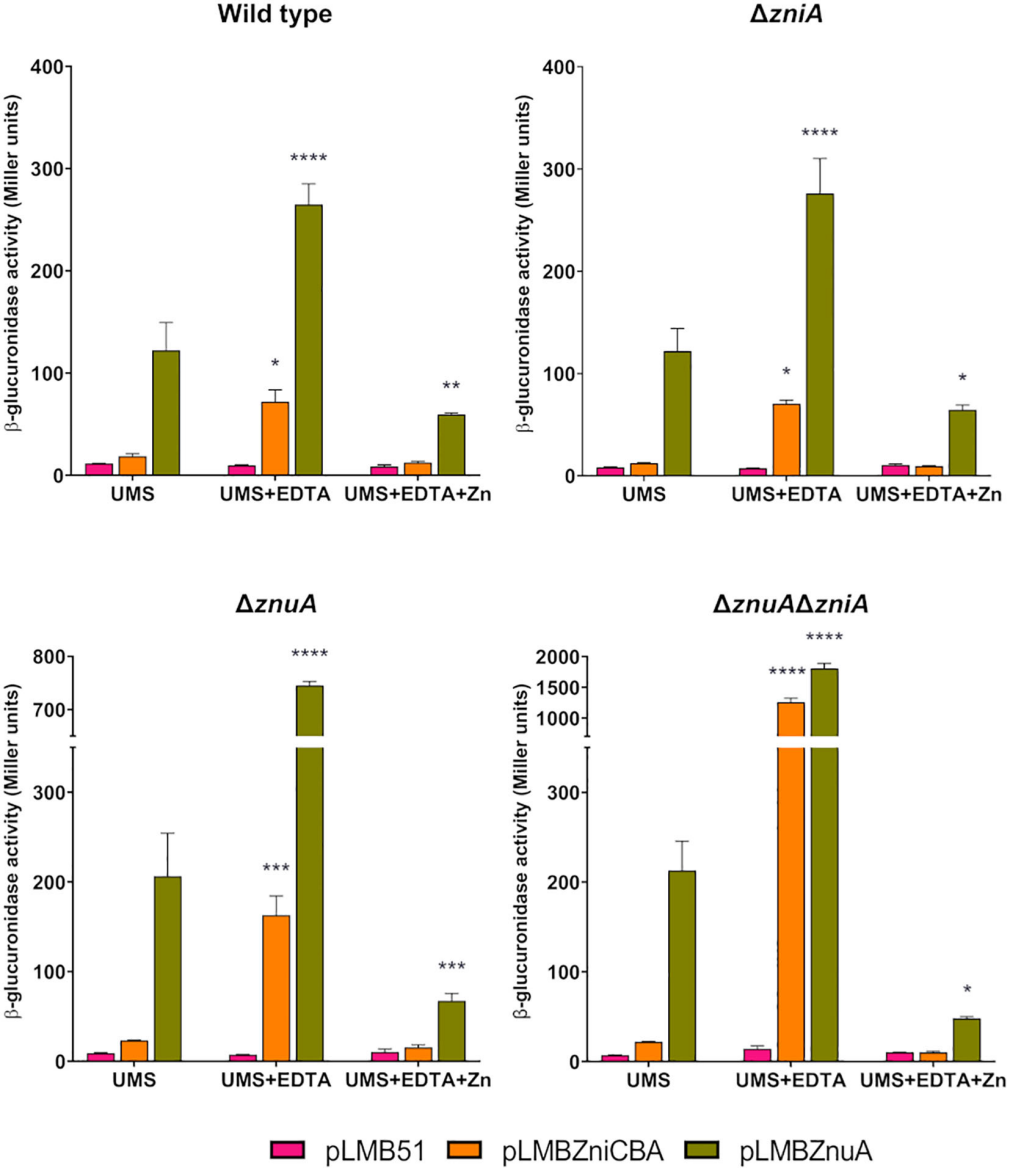
Expression analysis of *zniCBA* and *znuA* genes as a function of the zinc concentration in the culture medium. Rlv SPF25 (wild type) and its derivatives UPM1628 (Δ*znuA*), UPM1629 (Δ*zniA*), and UPM1630 (Δ*znuA*Δ*zniA*) strains harboring empty pLMB51 plasmid, pLMBZniCBA or pLMBZnuA reporter fusion plasmids were grown in UMS medium (UMS), in EDTA-chelated medium (50 µM EDTA; UMS+EDTA) or in the same medium supplemented with zinc (50 µM EDTA, 50 µM ZnSO_4_; UMS+EDTA+Zn). β-glucuronidase activities are the mean of two independent experiments with two replicates each ± standard error. Data were analyzed by one-way ANOVA and Dunnett´s test for multiple comparisons of means comparing reporter activities associated to each plasmid to those in UMS medium. *P < 0.05, **P < 0.01, ***P < 0.001, ****P < 0.0001.

We first analyzed *zni* gene expression under free-living conditions. To this aim, reporter activity was determined in bacterial cultures grown in UMS medium or in the same medium supplemented with EDTA (50 µM). In SPF25 and UPM1629 (Δ*zniA*) strains harboring the *zniCBA´-gusA* reporter fusion, only basal levels of enzymatic activity (*ca.* 20 units) were observed when cells were grown in UMS medium (**Figure 7**). In contrast, significant levels of activity were reported in both strains under metal-chelated conditions. Interestingly, under the same conditions, reporter activity increased in the *znuA*-deficient UPM1628 strain (*ca*. 8-fold over basal levels); finally, the expression of the reporter enzyme was highly induced (*ca*. 60-fold over basal levels) in the *znuA*/*zniA*-deficient UPM1630 strain. A similar pattern of results was observed when analyzing expression of the *znuA* gene through the use of *znuA*´-*gusA* fusion (**Figure 7**). In this case, the level of reporter activity was always higher than in the case of *zniCBA´-gusA* fusion, suggesting that ZnuA is the major transporter supporting Zn provision to the bacterium. In all cases, enzymatic activities associated with either reporter fusion were reduced to basal levels when the metal-chelated medium was supplemented with zinc (50 µM ZnSO_4_) in all the genetic backgrounds. These results confirm that the expression of both transporter systems is negatively regulated by zinc and that ZniCBA functions as an auxiliary transporter system in the absence of ZnuABC.

**Figure 8:**
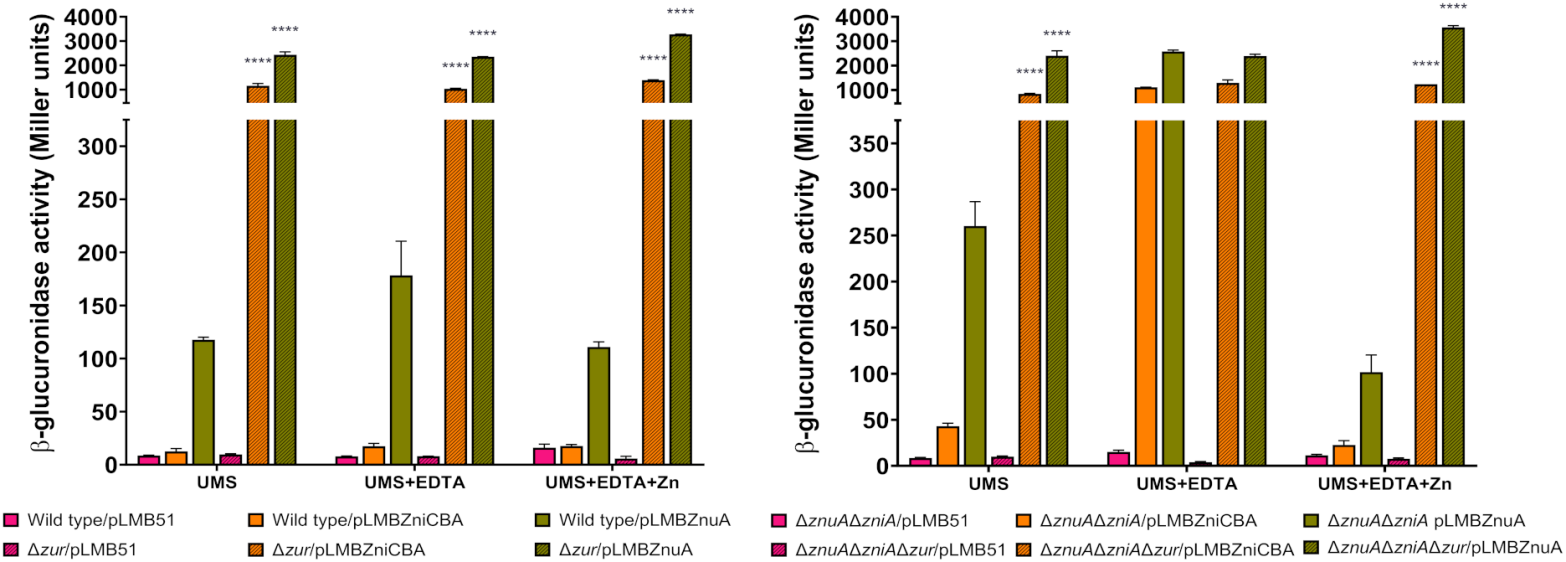
Analysis of Zur-dependent expression of *zniCBA* and *znuA* genes. Rlv SPF25 (wild type), UPM1630 (Δ*znuA*Δ*zniA*) and its *zur*-mutant derivatives UPM1633 (Δ*zur*) and UPM1634 (Δ*znuA*Δ*zniA*Δ*zur*) strains harboring empty pLMB51 plasmid, pLMBZniCBA or pLMBZnuA reporter fusion plasmids were grown in UMS medium (UMS), in EDTA-chelated medium (50 µM EDTA; UMS+EDTA) or in the same medium supplemented with zinc (50 µM EDTA, 50 µM ZnSO_4_; UMS+EDTA+Zn). β-glucuronidase activities are the mean of two independent experiments with two replicates each ± standard error. Data were analyzed by one-way ANOVA and Dunnett´s test for multiple comparisons of means comparing reporter activities associated to each plasmid between SPF25 or UPM1630 and its *zur*-deficient derivative strains at each condition assayed. ****P < 0.0001.

The identification of potential Zur-binding sites in the promoter region of the *zniCBA* operon and *znuA* gene (**Figure 1**) prompted us to investigate whether the expression of both systems is regulated by the transcriptional regulator Zur. To carry out these studies, pLMBZniCBA and pLMBZnuA plasmids were transferred into UPM1633 and UPM1634 strains (Δ*zur* and Δ*znuA*Δ*zniAΔzur* derivatives of SPF25 and UPM1630, respectively), and β-glucuronidase activity was determined following incubation in the different media. In these experiments, the levels of reporter activity were highly induced in all *zur*-deleted strains carrying pLMBZniCBA or pLMBZnuA plasmids, regardless the levels of ZnSO_4_ in the medium (**Figure 8**). These findings indicate that the *zniCBA* operon and *znuA* are negatively regulated by Zur.

**Figure 9:**
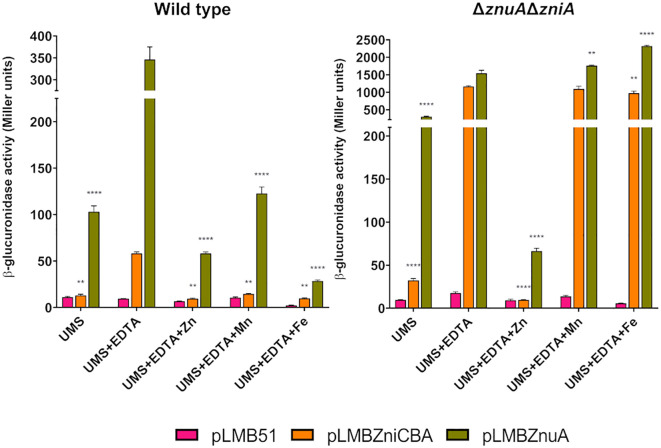
Effect of metal addition on *zniCBA* and *znuA* gene expression under metal-limiting conditions. Rlv SPF25 (wild type) and its derivative UPM1630 (Δ*znuA*Δ*zniA*) strain harboring empty pLMB51 plasmid, pLMBZniCBA or pLMBZnuA reporter fusion plasmids were grown in UMS medium (UMS), in EDTA-chelated medium (50 µM EDTA; UMS + EDTA) or in the same medium supplemented with 50 µM ZnSO_4_ (UMS + EDTA + Zn), MnSO_4_ (UMS + EDTA + Mn), or FeSO_4_ (UMS + EDTA + Fe). β-glucuronidase activities are the mean of two independent experiments with two replicates each ± standard error. Data were analyzed by one-way ANOVA and Dunnett´s test for multiple comparisons of means comparing reporter activities associated to each plasmid under each condition assayed to that in UMS + EDTA condition. **P < 0.01, ****P < 0.0001.

The metal specificity of responses of *zniCBA* and *znuA* was investigated in SPF25 and UPM1630 (Δ*znuA*Δ*zniA*) strains harboring pLMBZniCBA or pLMBZnuA plasmids through experiments similar to those described above, but adding other metal cations (Mn^2+^, Fe^2+^) instead of Zn^2+^ to EDTA-supplemented media (**Figure 9**). There was no effect of the addition of these metals in the case of double mutant UPM1630, indicating that the observed effect of Zn blocking was specific for this element. Interestingly, a significant repression of β-glucuronidase activity was shown when the same experiment was carried out in the wild-type background. This observation might be associated with a displacement of zinc-chelated to EDTA by these metals, thus decreasing the effective metal chelator concentration for the complexation of zinc in the medium, as previously suggested in *E*. *coli znu* regulation studies (Patzer and Hantke, 2000). This effect was not observed in UPM1630, likely because not enough zinc to repress the expression of the system enters into the cell in the absence of both transporter systems. Furthermore, a low but significant increment of the β-glucuronidase activity associated with the pLMBZnuA reporter plasmid was observed in UPM1630 strain grown in EDTA-chelated medium supplemented with manganese and iron. These results suggest that Rlv ZnuABC and ZniCBA are inducible, specifically by zinc starvation conditions, in a Zur-dependent manner.

**Figure 10:**
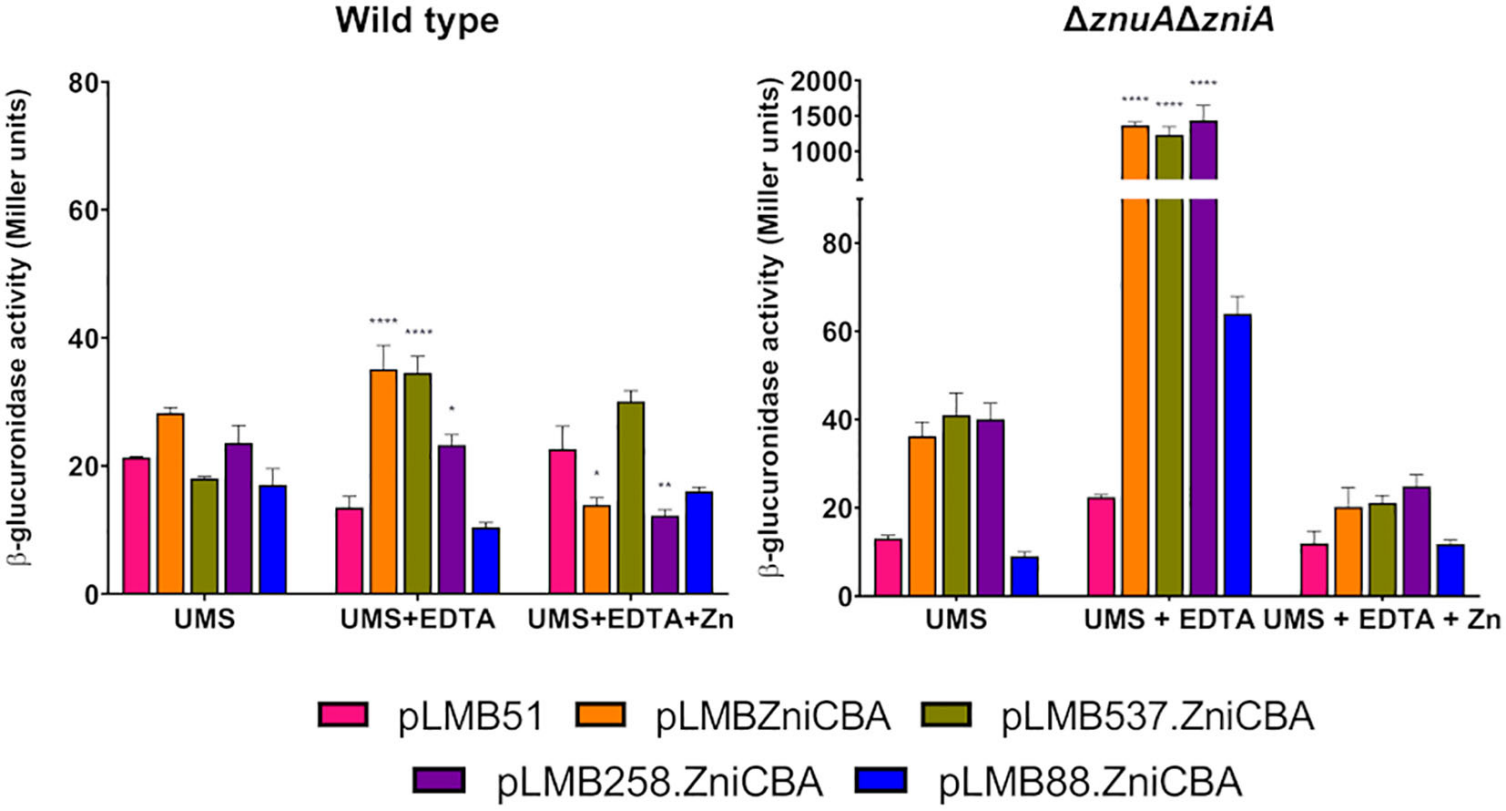
Deletion analysis of the promoter region of *zniCBA* operon under zinc-limiting conditions. Rlv SPF25 (wild type) and its derivative UPM1630 (Δ*znuA*Δ*zniA*) strains harboring empty pLMB51 plasmid, pLMBZniCBA, pLMB537.ZniCBA pLMB258.ZniCBA or pLMB88.ZniCBA reporter fusion plasmids were grown in UMS medium (UMS), in EDTA-chelated medium (50 µM EDTA; UMS+EDTA) or in the same medium supplemented with 50 µM ZnSO_4_ (UMS+EDTA+Zn). β-glucuronidase activities are the mean of two independent experiments with two replicates each ± standard error. Data were analyzed by one-way ANOVA and Dunnett´s test for multiple comparisons of means comparing reporter activities between each reporter plasmid and empty vector pLMB51 at each condition assayed. *P < 0.05, ** *P*< 0.01, ****P < 0.0001.

We also wanted to better define the regulatory region controlling the expression of the *zniCBA* operon. To this aim, a deletion analysis of its promoter region was carried out using transcriptional fusions to DNA regions of the promoter truncated 537, 258, and 88 bp upstream of the operon (plasmids pLMB537.ZniCBA, pLMB258.ZniCBA, and pLMB88.ZniCBA, respectively). The new plasmids, together with pLMBZniCBA, were independently transferred to wild-type (SPF25) and *zniA/znuA* double mutant (UPM1630) strains. Reporter activities were determined in bacterial cultures grown in UMS and in the same medium supplemented with the metal chelator EDTA with or without zinc (50 µM ZnSO_4_). In these experiments, fusions including the 537- and 258-bp fragments of the promoter region induced reporter activities that were similar to those associated with the original fusion spanning 775 bp (**Figure 10**). In contrast, strains carrying the shortest fragment (88 bp) induced only basal levels of reporter activity under all the conditions assayed. These observations indicate that a relevant regulatory region of the ZniCBA transporter system is located between positions 258 and 88 pb upstream of the first ATG codon of the operon, which is consistent with the presence of the Zur box in this region (**Figure 1B**).

### Analysis of *zniCBA* and *znuA* gene expression under symbiotic conditions

**Figure 11:**
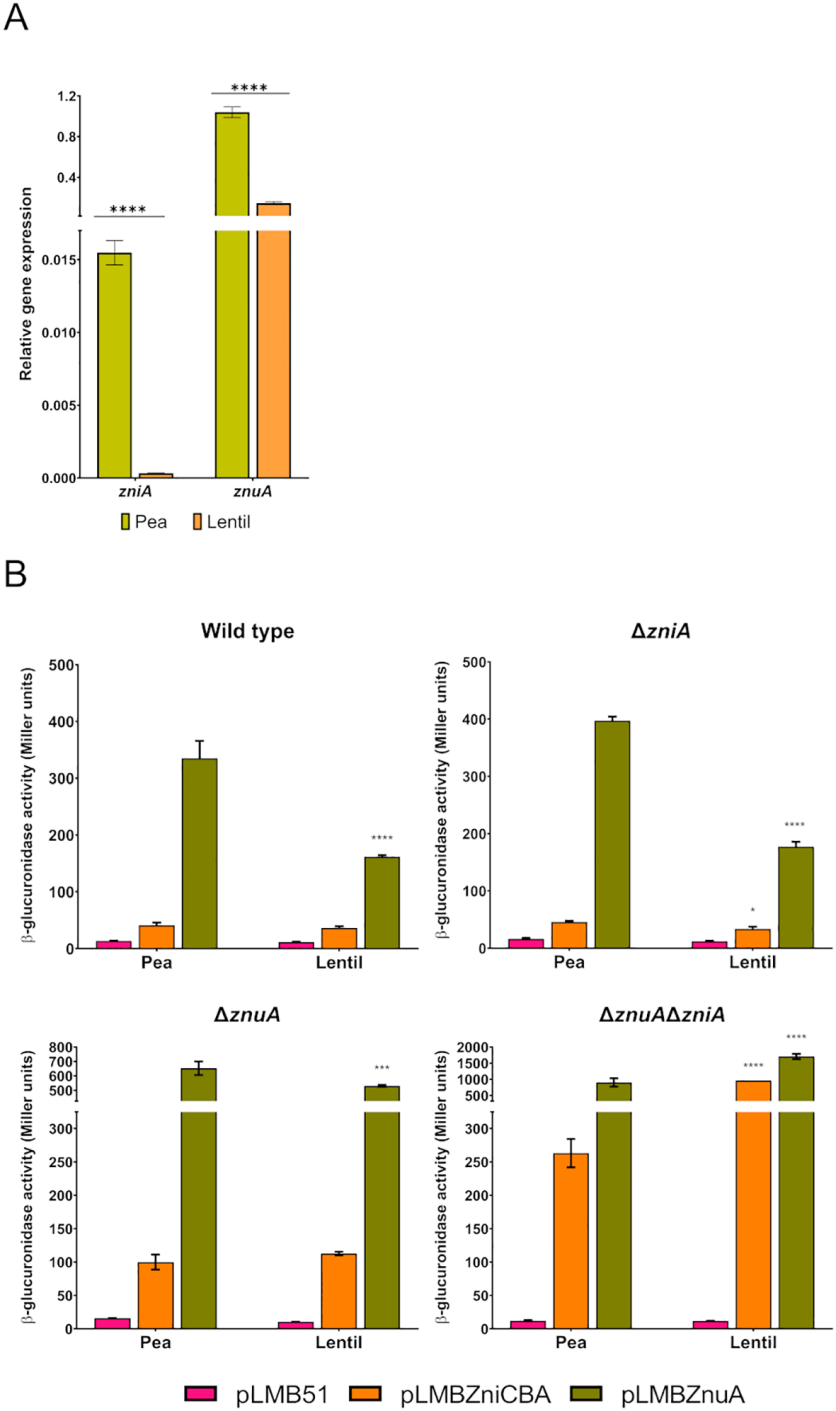
Symbiotic expression of *zniCBA* and *znuA* genes in pea and lentil nodules. **(A)** qRT-PCR analysis. Histogram shows the relative expression levels of *zniA* and *znuA* genes in bacteroids induced by Rlv SPF25 strain in pea and lentil nodules using the *rpoD* gene as standard for normalization. Plants were grown in a standard N-free nutrient solution (0.8 µM ZnSO_4_). Values are the means of three experimental replicates ± standard error. Data were analyzed by one-way analysis of variance (ANOVA) and Tukey test for multiple comparisons of means comparing expression levels between the two legume hosts. Values significantly different at *P* < 0.0001 (****) are indicated. **(B)** Reporter gene analysis of the expression of *zniCBA* and *znuA* genes. Histograms show β-glucuronidase activities of pea and lentil bacteroids induced by Rlv SPF25 (wild type) and its derivatives UPM1628 (Δ*znuA*), UPM1629 (Δ*zniA*), and UPM1630 (Δ*znuA*Δ*zniA*) strains harboring empty pLMB51 plasmid, pLMBZniCBA or pLMBZnuA reporter fusion plasmids as indicated. Plants were grown in a standard N-free nutrient solution (0.8 µM ZnSO_4_). Data are the mean of two independent experiments with two replicates each ± standard error. Data were analyzed by one-way ANOVA and Tukey test for multiple comparisons of means between the two legume host associated to each plasmid in each strain. *P < 0.05, ***P < 0.001, ****P < 0.0001.

Expression analysis of the *zniCBA* operon and *znuA* gene was also carried out under symbiotic conditions. We first analyzed by qRT-PCR whether the control of the host-dependent expression of *zniA* was regulated at the transcriptional level. This analysis was also extended to the *znuA* gene, as our proteomic analysis revealed that its encoded protein was detected in pea bacteroids but not in those developed in lentil plants (Durán et al., 2021). Quantification of the levels of transcripts revealed that both *zniA* and *znuA* genes were significantly upregulated in bacteroids from both pea and lentil (*ca*. 10-fold and 3-fold, respectively, for *zniA* and *znuA*) (**Figure 11A**). In this analysis, transcripts of the gene encoding the [NiFe] hydrogenase large structural subunit HupL, governed by a strong host-dependent control (Brito et al., 2008), exhibited expression increments of 100-fold in pea *versus* lentil bacteroids (20.2 ± 3.0 and 0.18 ± 0.02 relative expression levels in pea and lentil bacteroids, respectively). These data indicate that the host-dependent expression of *zniA* and *znuA* is controlled at the transcriptional level. In addition, these observations suggest that the metal provision to bacteroids might be more restrictive in pea than in lentil.

The symbiotic induction of *zniA* and *znuA* genes was also analyzed by using transcriptional fusions of *zniCBA* and *znuA* promoter regions to the *gusA* reporter gene in the pLMB51 plasmid. To this aim, we analyzed reporter activities in bacteroids prepared from plants grown in standard nutrient solution (0.8 µM ZnSO_4_) inoculated with SPF25 (wild type) and its derivatives UPM1628 (Δ*znuA*), UPM1629 (Δ*zniA*), and UPM1630 (Δ*znuA*Δ*zniA*) bearing transcriptional fusions for *zniCBA, znuA*, or just empty plasmid. Data revealed that expression levels of the reporter gene associated with *znuA* were higher than those related to *zniCBA* in all genetic backgrounds and in both legume hosts, which is consistent with qRT-PCR data (**Figure 11B**). Also, the symbiotic expression of the β-glucuronidase enzyme associated with both reporter fusion plasmids was higher in strains carrying the deletion in *znuA* (UPM1628) than in the wild type, and even higher levels of reporter activity were scored in the double mutant UPM1630, similar to what was previously observed in free-living cells. In addition, the activity profile recorded in a *zniA*-deleted mutant harboring pLMBZnuA plasmid increased in comparison to that in SPF25 in pea-bacteroids, suggesting compensatory levels of expression of this system in the absence of ZniA for the potential restriction to zinc in this legume. Enzymatic activities derived from both reporter fusion plasmids increased in lentil bacteroids induced by the UPM1630 strain, suggesting a higher level of zinc deprivation in this host in the absence of both transport systems.

## Discussion

In this work, we have characterized two zinc transporter systems, the canonical conserved ZnuABC and ZniCBA, that display similar, interchangeable functional roles. The presence of at least one of the systems is essential for Rlv SPF25 to grow under zinc-limiting conditions and for full symbiotic performance with pea and lentil plants. Bioinformatic analysis revealed high structural similarity of Rlv ZniA with other periplasmic zinc-binding proteins. The protein contains three conserved histidine residues also present in homologous ZniA from *K*. *pneumoniae*, AztC from *P*. *denitrificans*, and TroA from *A*. *tumefaciens.* The third conserved histidine residue (H193, Rlv ZniA numbering) correlates with Zn^2+^ as a ligand of the binding protein, whereas the presence of a glutamate residue at the same position has been associated with Fe^2+^ or Mn^2+^ specificity (Claverys, 2001; Hantke, 2001; Blindauer, 2015). Homology modelling of the Rlv ZniA structure confirms that its metal substrate might be coordinated by these histidines and a glutamate residue (E268, Rlv ZniA numbering) since they occupy the same position in the metal binding site of *K*. *pneumoniae* ZniA (Maunders et al., 2024). This is also in line with our results on ZniA site-directed mutant analysis revealing that the conserved histidines are essential for the function of the ZniCBA transporter system under zinc-limiting conditions. In addition, Rlv ZniA does not contain the central histidine domain present in AztC and TroA that has also been associated with zinc specificity (Blindauer, 2015).

Functional evidence for a direct involvement of Rlv ZniA in zinc transport has been established in this work by the rescue of UPM1630 by zinc addition. The reversion of the double mutant phenotype by expressing either ZniA or ZnuA suggests that ZnuABC and ZniCBA play redundant roles under these conditions. It has been reported that, in addition to ZnuABC, some bacterial species express a second zinc transporter playing similar and redundant functions supporting growth under zinc-limiting conditions (Desrosiers et al., 2010; Corbett et al., 2012; Plumptre et al., 2014; Pederick et al., 2015; Handali et al., 2015; Neupane et al., 2019; Maunders et al., 2024). These transporter systems might play an essential role in bacteria adaptation to environments with low zinc availability, as previously suggested (Neupane et al., 2019). Interestingly, Rlv ZniA did not revert the defective phenotype of a *znuA zniCBA* mutant, indicating that ZniA does not exert its functional role through a crosstalk with ZnuB permease. This is consistent with the low sequence identity between both SBPs (26.04%) and strongly supports the high affinity zinc uptake contribution of Rlv ZniCBA under extreme zinc-limiting conditions. Similar results were observed in *P*. *denitrificans*, in which a specificity in the interaction between the SBP and its cognate permease from ZnuABC and AztABCD transporter systems was also demonstrated (Meléndez et al., 2020). In contrast, the *Streptococcus pneumoniae* AdcABC transporter system, the homologue to ZnuABC in Gram-positive bacteria, shares the permease AdcB and ATPase AdcC with the orphan SBP AcdII, with both SBPs contributing to the growth of the bacteria under zinc-limiting conditions (Bayle et al., 2011). The expression levels of ZniCBA were lower than ZnuA but high enough to support the growth at wild-type levels in the absence of ZnuABC, as previously observed with the AztABCD system in *P*. *denitrificans* (Handali et al., 2015). Since ZniCBA upregulation in response to the metal chelator EDTA increases in the absence of ZnuA, ZniCBA might function as a backup system for high-affinity zinc uptake to ensure adequate metal levels in the cell.

Reporter gene analyses have shown that the expression of *zniCBA* and *znuA* genes is dependent on zinc levels in a Zur-dependent manner in free-living cells. The transcriptional regulator Zur controls the expression of many zinc uptake transporters, repressing their synthesis in the presence of zinc (Mikhaylina et al., 2018). In our system, deletion of *zur* greatly increases *zni* and *znu* expression, consistent with the presence of a Zur box in their promoter regions. Furthermore, promoter deletion analysis showed that removal of the Zur box-like sequence also affects the regulation of *zniCBA* expression. The reduced levels of *zniCBA* expression when using the shortest form of the promoter suggest that, in addition to the Zur box, the 257–88 upstream region might contain other regulatory signals related to the transcription of *zniCBA* genes.

Analysis of metal specificity of the transcriptional repression in EDTA-chelated medium revealed that in the absence of both SBPs, Zur responds specifically to zinc, supporting the idea that both transporter systems are involved in zinc acquisition. The higher derepression of the ZnuABC system in the presence of Mn^2+^ or Fe^2+^ might be accounted for a competitive inhibition of Zn^+2^ uptake by an unspecific transporter, as previously stated (Patze and Hantke, 2000). Our results also showed that ZniCBA and ZnuA responded to Mn^2+^ and Fe^2+^ in a Zur-dependent manner in the wild-type strain. Previous studies reported that *A. tumefaciens* ZnuA and TroC repressed its EDTA-induced expression in the presence of these metals, accounting for zinc for the highest capacity (Bhubhanil et al., 2014; Chaoprasid et al., 2016). Furthermore, *S. fredii* ZnuA was also observed to respond to cobalt in a Zur-dependent manner that was explained by a mismetalation of cobalt to Zur (Zhang et al., 2020). *In vitro* analysis of metal binding to Zur has revealed the formation of a Zur-DNA complex with manganese in addition to zinc (Gaballa and Helmann, 1998; Patzer and Hantke, 2000; Schröder et al., 2010) and a correlation between metal sensor K_DS_ and cytosolic-free concentration has been reported (Osman et al., 2017). In our system, a mismetalation of manganese and iron to Zur would not explain the repression pattern observed in the absence of ZniA and ZnuA in the UPM1630 strain. Our hypothesis is that an excess of these metals in the cell leads to a displacement of zinc chelated by EDTA to free zinc, which might be transported inside, bind to Zur, and thus repress the expression of the systems. This might happen in the wild-type strain, where these low amounts of free zinc would be internalized by the transporters, but not in the UPM1630 strain, where the Zni and Znu systems are not active and extracellular zinc content might not be enough to repress their expression in a Zur-dependent manner.

We have demonstrated that ZnuABC and ZniCBA are required for the Rlv-pea and -lentil symbiosis. Mutation of both SBPs led to a drastic reduction of symbiotic performance in pea and lentil plants, indicating that both transporter systems are involved in the adaptation and survival of Rlv in pea and lentil nodules. The defective symbiotic phenotype of the double mutant strain Rlv UPM1630 was restored by the expression of ZniA or ZnuA, indicating that pea and lentil bacteroids induced by this strain are affected in zinc uptake. Previous studies have shown that the contribution of ZnuA to the symbiotic performance of *S. fredii* depends on the legume host. A significant decrease in shoot dry weight was observed in *Glycine max* and *Cajanus cajan* plants inoculated with the CCBA45436 strain, but not in *Glycine soja* (Jiao et al., 2018; Zhang et al., 2020). These results suggest that alternative zinc uptake systems are operating, governed by the legume host. As this strain lacks an Rlv ZniCBA-like transporter system, alternative zinc transporters might not supply enough zinc for CCBA45436 strain symbiotic performance in *G*. *max* or *C. cajan* plants where the metal availability might be lower in the nodule. In addition, it was observed a higher accumulative contribution of ZnuA and accessory zinc uptake proteins (Zip1, Zip2, and c06450) to nodulation of this strain in *G*. *soja* and *C*. *cajan* but not in *G*. *max* (Zhang et al., 2020). Our results indicate that zinc is not entering into the bacteroids induced by Δ*znuA*Δ*zniA* or, alternatively, that the zinc supplied by potential alternative metal transporters is not enough to inhibit the Zur-dependent expression of both systems. These results align with the observation that the defective symbiotic phenotype of UPM1630 was not rescued with a nutrient solution containing zinc concentrations 100 times higher (80 µM ZnSO_4_) than those of the standard nutrient solution (0.8 µM ZnSO_4_) in both legumes (**Figure 6**). Furthermore, even in the presence of zinc levels 1000-fold higher than in the normal nutrient solution, the double mutant UPM1630 was unable to obtain zinc, neither to correct the deficient plant growth (data not shown) nor to repress transcription of *zni/znu* genes (**Supplementary Figure S4**). This “impermeability” to Zn in zinc transporter mutants has not been observed in other symbiotic systems, such as the *Sinorhizobium*/*Glycine max* system, where mutations in zinc transporters were complemented for nodulation by the addition of 700 µM ZnSO_4_ (Zhang et al., 2020).

Our analysis has shown that expression levels of ZniCBA and ZnuA increase in the absence of ZnuA, and both transporter systems are highly induced in bacteroids from pea and lentil nodules induced by a Δ*zniA*Δ*znuA* mutant strain. These data support the hypothesis that ZniCBA functions as an auxiliary zinc transporter system under these conditions. Although the levels of ZniCBA expression were also lower than ZnuA under symbiotic conditions, they were high enough to optimize the symbiotic performance of Rlv with the legume host. Consistent with our results, the presence of more than one zinc transporter system has been extensively reported to allow bacterial survival under conditions in which the availability of zinc is low. Previous studies revealed that the levels of expression of *P*. *denitrificans* ZnuA were higher than those of the SBP AztC in bacterial cultures grown under zinc-limiting conditions with redundant functional roles (Handali et al., 2015). In addition, in *A*. *tumefaciens*, the cooperation of TroCBA, ZnuABC, and two zinc chaperones present in this system (ZinT and YciC) are required for growth in zinc-depleted conditions (Chaoprasid et al., 2016). In *K*. *pneumoniae*, although ZniA is expressed at higher levels than ZnuA under zinc-limiting conditions, both SBPs were required for bacterial virulence and pathogenicity (Maunders et al., 2024).

The results show a host-dependent expression of both zinc transporter systems exerted at transcriptional levels. qRT-PCR analysis reveals that both transporters are expressed at lower levels in lentil bacteroids induced by the wild-type strain, whereas their expression increases in pea bacteroids, suggesting a higher restriction for zinc uptake into pea bacteroids. Similar results were obtained with the ZnuA-homologue in *S. fredii* CCBAU45436, which was shown to be differentially upregulated in different soybean species, with higher levels of expression in bacteroids from *G. max* than in *G. soja* (Jiao et al., 2018). Preliminary analysis by ICP-MS indicates that the zinc content of plant cytosol from pea and lentil nodules induced by the Rlv SPF25 strain is similar (data not shown). This suggests that the host-dependent pattern of both transporter systems might be explained not by differences in total zinc content but rather by the presence of zinc complexes that could be different in both symbioses, and this condition might be responsible for a higher restriction in providing zinc to the bacteroid in pea *versus* lentil pants. This is in line with our previous studies that showed that nickel is differentially chelated by malate and citrate in the cytoplasm of nodules induced by Rlv UPM791 in different legumes (Cacho et al., 2010; our unpublished results), an observation associated to the strong effects of the legume host on the Rlv UPM791 [NiFe]-hydrogenase enzyme, along with the different phenotypes of defective mutants in nickel transporters in pea and lentil plants (Brito et al., 2010). Analysis of the molecular basis of such host-dependent effect can allow further advances in the understanding of bacterial adaptation to this fascinating plant-microbe interaction.

## Data Availability

The original contributions presented in the study are included in the article/**Supplementary Material**. Further inquiries can be directed to the corresponding author.
