# Supplementary material for: Two zinc ABC transporters contribute to Rhizobium leguminosarum symbiosis with Pisum sativum and Lens culinaris
Source: Front Plant Sci. 2025 Jun 9;16:1598744. doi: 10.3389/fpls.2025.1598744 (PMC12183173; doi:10.3389/fpls.2025.1598744)
Supplement: Supplementary file 1 [file DataSheet1.docx]

**Supplementary Figure 1. Phylogenetic tree of Zn/Mn-binding proteins of bacterial ABC transporter systems**. Phylogenetic tree of sequences of Zn/Mn proteins were performed with MEGA12 (Tamura *et al*., 2012) using a Neighbour-Joining algorithm method (Saitou and Nei, 1987). Only bootstrap values greater than 75% (calculated for 1000 subsets) are shown (Felsenstein,1985). The scale bar indicates number of substitutions per site. Sequence names shown in the tree contain the abbreviated name of the bacterial species followed by the name of the protein and the accession numbers from GenBank. The substrate of the functionally characterized metal-binding protein and the reference of the study are indicated. Bacterial strains: RlvUPM791, *Rhizobium leguminosarum* bv. viciae UPM791; Kpn, *Klebsiella pneumoniae* AJ218; Atu, *Agrobacterium tumefaciens* C58; Pde, *Paracoccus denitrificans* PD1222; Tpa, *Treponema pallidum* Nichols; Spn, *Streptococcus pneumoniae* D39; Syn, *Synechocystis* sp. PCC 6803; Sme, *Sinorhizobium meliloti* 1021; Rlv3841, *R. leguminosarum* bv. viciae 3841; Eco, *Escherichia coli* K-12 substrain MG1655; Sfre: *Sinorhizobium fredii* CCBAU45436.

**Supplementary Figure 2.** **Sequence alignment of RLV_3444 and Zn-binding proteins of bacterial ABC transporter systems**. The areas shaded in yellow highlight the conserved residues, while the bar over the alignment illustrates the degree of conservation (deep blue, no conservation; red, high degree of conservation). Asterisks indicate conserved and metal coordination residues identified in *K. pneumoniae* ZniA and *P. denitrificans* AztC crystal structures. The box denotes the histidine-rich tract present in *A*. *tumefaciens* TroA and *P. denitrificans* AztC. The vertical arrow shows the predicted cleavage site for a signal peptidase (SignalP 5.0 tool). RLV_3444: RLV_3444 from *R*. *leguminosarum* bv. viciae UPM791 (AVC48609.1); Atu_TroA: TroA from *A. tumefaciens* C58 (AAK90208.1); Kpn_ZniA: ZniA from *K*. *pneumoniae* AJ218 (WP_023286807.1); Pde_AztC: AztC from *P. denitrificans* PD1222 (WP_011747896.1).

**Supplementary Figure 3. Effect of the expression of *zniCBA* operon on the growth of Rlv UPM1632 strain under metal limiting conditions.** Rlv strains were grown until the stationary phase in zinc-depleted (no zinc added) UMS medium (UMS), in EDTA-chelated medium (no zinc added, 50 µM EDTA; UMS+EDTA) or in the same medium supplemented with 50 µM ZnSO_4_ (UMS+EDTA+Zn). Each OD determination represents the mean of three replicates ± standard error. All standard errors were below 10%. Strains: wild type (SPF25, pLMB51), Δ*znuA*Δ*zniCBA* (UPM1632, pLMB51). *zniCBA* was provided cloned in pLMB51 plasmid as indicated (pLMcZni plasmid).

**Supplementary Figure 4. Symbiotic expression of *zniCBA* and *znuA* genes as a function of the zinc concentration in the plant growth nutrient solution**. Histograms show the reporter gene analysis of the expression of *zniCBA* and *znuA* genes in pea bacteroids induced by Rlv SPF25 (wild type), UPM1628 (Δ*znuA*), UPM1629 (Δ*zniA*) and UPM1630 (Δ*znuA*Δ*zniA*) strains harbouring empty pLMB51 plasmid, pLMBZniCBA or pLMBZnuA reporter fusion plasmids as indicated. Plants were grown in a standard N-free nutrient solution (0.8 µM ZnSO_4_) or in same solution containing 800 µM ZnSO_4_ as indicated. Data are the mean of two independent experiments with two replicates each ± standard error. Data were analysed by one-way ANOVA and Tukey test for multiple comparisons of means between the two zinc concentrations associated to each plasmid in each strain. ** *P* < 0.01, **** *P* < 0.0001.

**Supplementary Table 1.** Bacterial strains and plasmids used in this work.

| **Strain or plasmid** | **Relevant genotype or phenotype** | **Source or reference** |
| --- | --- | --- |
| *Rhizobium leguminosarum* |  |  |
| UPM791 | 128C53 wild type; Str^r^ Nod^+^ Fix^+^ Hup^+^ | Leyva *et al*., 1987 |
| SPF25 | UPM791 with P*fixN*::*hupSL* | Brito *et al*., 2002 |
| UPM1628 | SPF25 Δ*znuA* | This work |
| UPM1629 | SPF25 Δ*zniA* | This work |
| UPM1630 | SPF25 Δ*znuA*Δ*zniA* | This work |
| UPM1631 | SPF25 Δ*zniCBA* | This work |
| UPM1632 | SPF25 Δ*znuA*Δ*zniCBA* | This work |
| UPM1633 | SPF25 Δ*zur* | This work |
| UPM1634 | UPM1630 Δ*zur* | This work |
| *Escherichia coli* |  |  |
| DH5_α_ | *recA1 endA1 gyrA96 thi hsdR17 supE44 relA1* Δ(*lacZYA*-*argF*)*U169* (Φ80d*lacZ*ΔM15) *deoR phoA* | Hanahan, 1983 |
| S17.1 | *thi pro hsdR^-^ hsdM^+^ recA* RP4::2-Tc::Mu-Kan::T7, Spec^r^ Str^r^ | Simon, Priefer and Pülher, 1983 |
| Plasmids |  |  |
| pCR2.1-TOPO | PCR product cloning vector; Amp^r^ , Kan^r^ | Invitrogen |
| pBlueScript-II KS+ | PCR product cloning vector; Amp^r^ | Stratagene |
| pK18*mobsacB* | pK18 derivative *sacB*; *lacZ* mob, Kan^r^ | Schäfer *et al.*, 1994 |
| pK18.ZnuA | pK18*mobsacB* with a deletion in *znuA*; Kan^r^ | This work |
| pK18.ZniA | pK18*mobsacB* with a deletion in *zniA*; Kan^r^ | This work |
| pK18.ZniCBA | pK18*mobsacB* with a deletion in *zniCBA*; Kan^r^ | This work |
| pK18.Zur | pK18*mobsacB* with a deletion in *zur*; Kan^r^ | This work |
| pLMB51 | *gusA* reporter gene vector with taurine-dependent promoter, Tet^r^ | Tett *et al.*, 2012 |
| pLMBZniCBA | pLMB51 derivative containing 775 bp upstream of *zniC* | This work |
| pLMB537.ZniCBA | pLMB51 derivative containing 537 bp upstream of *zniC* | This work |
| pLMB258.ZniCBA | pLMB51 derivative containing 258 bp upstream of *zniC* | This work |
| pLMB88.ZniCBA | pLMB51 derivative containing 88 bp upstream of *zniC* | This work |
| pLMBZnuA | pLMB51 derivative containing *znuA* promoter region | This work |
| pLMBcZni | pLMB51 derivative containing *zniCBA* operon and its promoter | This work |
| pBBR1MCS-2 | Broad-host-range vector with a P*lac* promoter, Kan^r^ | Kovach *et al.*, 1995 |
| pBBRZniA | pBBR1MCS-2 derivative containing *zniA* and 258 bp upstream of *zniC* gene | This work |
| pBBRZnuA | pBBR1MCS-2 derivative containing *znuA* and its promoter | This work |
| pBBRZniA_ST_ | pBBR1MCS-2 derivative containing *zniA* gene fused to a *Strep*-tag II coding sequence in its 3´-end and 775 bp upstream of *zniC* gene | This work |
| pBBRZniA.H62A_ST_ | pBBRZniA_ST_ derivative, *zniA_st_* H62A | This work |
| pBBRZniA.H127A_ST_ | pBBRZniA_ST_ derivative, *zniA_st_* H127A | This work |
| pBBRZniA.H193A_ST_ | pBBRZniA_ST_ derivative, *zniA_st_* H193A | This work |

**Supplementary Table 2.** Primers used in this work

| **Primer** | **Sequence (5´-3´)** | **Use** |
| --- | --- | --- |
| P1_rlv_3444_BamHI | AAAGGATCCGAATATCGATCTCCTGCATGT | *zniA* deletion |
| P2_rlv_3444 | CCCCCGGGGGCCCCCCGAAGTCTCCTTGGGATCAG |  |
| P3_rlv_3444 | GGGGGCCCCCGGGGGTCGAGCTGAGGATCGAGAGG |  |
| P4_rlv_3444_HindIII | AAAAAGCTTCTCTCCCGGCTTTCGCCGCT |  |
| P1_rlv_3442-44_BamHI | AAAGGATCCGTCACTGATGCGGCACCTCA | *zniCBA* deletion |
| P2_ rlv_3442-44 | CCCCCGGGGGCCCCCCAACTTGCCGATTTCATCGCT |  |
| P3_ rlv_3444 | GGGGGCCCCCGGGGGTCGAGCTGAGGATCGAGAGG |  |
| P4_rlv_3444_HindIII | AAAAAGCTTCTCTCCCGGCTTTCGCCGCT |  |
| P1_ZnuA_BamHI | AAAGGATCCGCAGATCGGGCTTGCGGGCA | *znuA* deletion |
| P2_ZnuA | CCCCCGGGGGCCCCCCAACATGCTCCGCTTGAATT |  |
| P3_ZnuA | GGGGGCCCCCGGGGGGACTCTCATGCGCGCTCCGC |  |
| P4_ZnuA_HindIII | AAAAAGCTTACGGTCACCATGGTGTCGAG |  |
| P1_Zur | CTGTTCAGGCAGGCGATGTA | *zur* deletion |
| P2_Zur | CCCCCGGGGGCCCCCGCGGCGTGATCGGTTCAGGT |  |
| P3_Zur | GGGGGCCCCCGGGGGCGTCATGAGCCTTGTCCTTG |  |
| P4_zur | GCTGGCGATCGGCCTCGTCA |  |
| Prom1_ rlv_3442-44_BamHI_F | TTTGGATCCCTTTTCATTTCCCTTTGCGT | Generation of pLMBZniCBA,  pLMB537.ZniCBA,  pLMB258.ZniCBA, pLMB88.ZniCBA plasmids |
| Prom2_ rlv_3442-44_BamHI_F | TTTGGATCCGGCCCGATGATTGCGCTGAA |  |
| Prom3_ rlv_3442-44_BamHI_F | TTTGGATCCGCACGCACGCGATCATCTCA |  |
| Prom4_ rlv_3442-44_BamHI_F | TTTGGATCCTGGGACAGTCGGCTTTATCC |  |
| Prom5_ rlv_3442-44_XbaI_R | TTTTCTAGAGGACGGCGGGGTGCCTGTTA |  |
| Prom_ZnuA_BamHI_F | TTTGGATCCCCTGATACCAGCGGCGGTCT | Generation of pLMBZnuA plasmid |
| Prom_ZnuA_XbaI_R | TTTTCTAGATGCGAGGGTGGGCATTCTGA |  |
| Prom3_ rlv_3442-44_BamHI_F | TTTGGATCCGCACGCACGCGATCATCTCA | Generation of pBBRZniA plasmid |
| P2_Com_rlv_3444 _R | CCCCCGGGGGCCCCCCTCAGACGGCGGGGTGCCTGTTA |  |
| P3_Com_rlv_3444 _F | GGGGGCCCCCGGGGGTCCCAAGGAGACTTCGATGA |  |
| P4_Com_rlv_3444_ XbaI_R | TTTTCTAGACCTCAGCTCGATTTCGCCAT |  |
| Prom_ZnuA_BamHI_F | TTTGGATCCCCTGATACCAGCGGCGGTCT | Generation of pBBRZnuA plasmid |
| Com_ZnuA_XbaI_R | TTTTCTAGAAGTCATGTGCTGGAGAGGCAGTC |  |

**Supplementary Table 2.** Primers used in this work (*Continued)*

| **Primer** | **Sequence (5´-3´)** | **Use** |
| --- | --- | --- |
| Prom3_ rlv_3442-44_BamHI_F | TTTGGATCCGCACGCACGCGATCATCTCA | Generation of pBBRZniA_ST_ plasmid |
| P2_Com_rlv_3444 _R | CCCCCGGGGGCCCCCCTCAGACGGCGGGGTGCCTGTTA |  |
| P3_Com_rlv_3444 _F | GGGGGCCCCCGGGGGTCCCAAGGAGACTTCGATGA |  |
| P4_rlv_3444strep_XbaI_R | TTTTCTAGAGCTCACTTTTCGAACTGCGGGTGGCTCCAGCTAGCGCTCGATTTCGCCATGGCGGCG |  |
| Prom1_ rlv_3442-44_BamHI_F | TTTGGATCCCTTTTCATTTCCCTTTGCGT | Generation of pLMBcZni plasmid |
| rlv_3442-44_XbaI_R | AAATCTAGATCAGCTCGATTTCGCCATGG |  |
| rlv_3444_H62A_F | GAACGGCGATCCCGCCGAATTCGAGCCGT | Site-directed mutagenesis of *zniA_ST_* |
| rlv_3444_H62A_R | ACGGCTCGAATTCGGCGGGATCGCCGTTC |  |
| rlv_3444_H127A_F | AATCACCGATCCGGCTGTCTGGAACAGCC |  |
| rlv_3444_H127A_R | GGCTGTTCCAGACAGCCGGATCGGTGATT |  |
| rlv_3444_H193A_F | GGTGCTGACCAGCGCCGATGCCTTCGGTT |  |
| rlv_3444_H193A_R | AACCGAAGGCATCGGCGCTGGTCAGCACC |  |
| rlv_3444_ qPCR_R | ATCAAGGGCGAGCATGTGAA | qRT-PCR |
| rlv_3444_ qPCR_R | ATTCGACATAGAGCTCGCCG |  |
| znuA_ qPCR_R | CCGCCAATGCACTGACCTA |  |
| znuA_ qPCR_R | ACCGGTGCTCGAAATACTGG |  |
| rpoD_ qPCR_F | GCTTCGACCATTTCCTTCTTGG |  |
| rpoD_ qPCR_R | GATGAAGTCGATCGGAATCTG |  |
| hupL_qPCR_F | AGAATGGCTACTGGGGCAAC |  |
| hupL_qPCR_R | CCATCGACGTTGATCGGACA |  |

**Supplementary Table S3.** *R. leguminosarum* RLV_3444 Dali analysis results.

| **Rank** | **PDB Code** | **Z-score** | **rmsd** | **% ID** | **Protein description** | **Cation bound** |
| --- | --- | --- | --- | --- | --- | --- |
| 1 | 8svc | 39.7 | 1.2 | 57 | *Klebsiella pneumoniae* metal ABC transporter substrate-binding protein | Zn^2+^ |
| 2 | 5w57 | 39.4 | 1.3 | 42 | *Paracoccus denitrificans* high-affinity zinc uptake system protein AztC | Zn^2+^ |
| 3 | 5i4k | 37.8 | 1.5 | 31 | *Listeria monocytogenes* manganese-binding lipoprotein MntA | Mn^2+^ |
| 4 | 1xvl | 36.9 | 1.5 | 31 | *Synechocystis sp* manganese transporter MntC | Mn^2+^ |
| 5 | 7me2 | 36.6 | 1.5 | 30 | *Yersinia pestis* iron-binding protein YfeA | Fe^2+^ |
| 6 | 3hh8 | 36.6 | 1.8 | 28 | *Streptococcus pyogenes* iron ABC transporter substrate-binding lipoprotein MtsA | Fe^2+^ |
| 7 | 4k3v | 36.1 | 1.8 | 28 | *Staphylococcus aureus* manganese transporter MntC | Mn^2+^ |
| 8 | 1psz | 36.0 | 1.9 | 26 | Pneumococcal surface antigen PSAA | Zn^2+^ |
| 9 | 4oxq | 35.3 | 1.9 | 26 | *Staphylococcus pseudintermedius* metal-binding protein SitA | Zn^2+^ |
| 10 | 1k0f | 34.9 | 1.9 | 32 | *Treponema pallidum* zinc-binding protein TroA | Zn^2+^ |

Rank denotes the positions of the 10 proteins with the best structural homology to the predicted mature RLV_3444 (25-299 residues) related to all structures available in the PDB. Z-scores higher than 20 denotes that proteins are likely to be structural homologues. rmsd.: root mean square deviation of each structural alignment; % ID: percentage of sequence identity. The cation bound in the corresponding crystalline structures is indicated.

**REFERENCES**

Bartsevich, V. Y., and Pakrasi, H. B. (1996). Manganese transport in the cyanobacterium *Synechocystis* sp. PCC 6803. *J. Biol. Chem.* 271, 26057–26061. doi: 10.1074/jbc.271.42.26057

Bayle, L., Chimalapati, S., Schoehn, G., Brown, J., Vernet, T., and Durmort, C. (2011). Zinc uptake by *Streptococcus pneumoniae* depends on both AdcA and AdcAII and is essential for normal bacterial morphology and virulence. *Mol. Microbiol.* 82, 904–916. doi: 10.1111/j.1365-2958.2011.07862.x

Bhubhanil, S., Sittipo, P., Chaoprasid, P., Nookabkaew, S., Sukchawalit, R., and Mongkolsuk, S. (2014). Control of zinc homeostasis in *Agrobacterium tumefaciens* via zur and the zinc uptake genes *znuABC* and *zinT*. *Microbiology (United Kingdom)* 160, 2452–2463. doi: 10.1099/mic.0.082446-0

Brito, B., Palacios, J. M., Imperial, J., and Ruiz-Argüeso, T. (2002). Engineering the *Rhizobium leguminosarum* bv. viciae hydrogenase system for expression in free-living microaerobic cells and increased symbiotic hydrogenase activity. *Appl. Environ. Microbiol.* 68, 2461–2467. doi: 10.1128/AEM.68.5.2461-2467.2002

Chaoprasid, P., Dokpikul, T., Johnrod, J., Sirirakphaisarn, S., Nookabkaew, S., Sukchawalit, R., et al. (2016). *Agrobacterium tumefaciens* Zur regulates the high-affinity zinc uptake system TroCBA and the putative metal chaperone YciC, along with ZinT and ZnuABC, for survival under zinc-limiting conditions. *Appl. Environ. Microbiol.* 82, 3503–3514. doi: 10.1128/AEM.00299-16

Desrosiers, D. C., Sun, Y. C., Zaidi, A. A., Eggers, C. H., Cox, D. L., and Radolf, J. D. (2007). The general transition metal (Tro) and Zn^2+^ (Znu) transporters in *Treponema pallidum*: Analysis of metal specificities and expression profiles. *Mol. Microbiol.* 65, 137–152. doi: 10.1111/j.1365-2958.2007.05771.x

Dintilhac, A., Alloing, G., Granadel, C., and Claverys, J. P. (1997). Competence and virulence of *Streptococcus pneumoniae*: Adc and PsaA mutants exhibit a requirement for Zn and Mn resulting from inactivation of putative ABC metal permeases. *Mol. Microbiol.* 25, 727–739. doi: 10.1046/j.1365-2958.1997.5111879.x

Felsenstein, J. (1985). Confidence limits on phylogenies: an approach using the bootstrap. *Evolution* 39, 783–791. doi: 10.1111/j.1558-5646.1985.tb00420.x.

Hanahan, D. (1983). Studies on transformation of *Escherichia coli* with plasmids. *J. Mol. Biol.* 166, 557–580. doi: 10.1016/S0022-2836(83)80284-8

Hazlett, K. R. O., Rusnak, F., Kehres, D. G., Bearden, S. W., La Vake, C. J., La Vake, M. E., et al. (2003). The *Treponema pallidum* *tro* operon encodes a multiple metal transporter, a zinc-dependent transcriptional repressor, and a semi-autonomously expressed phosphoglycerate mutase. *J. Biol. Chem.* 278, 20687–20694. doi: 10.1074/jbc.M300781200

Hood, G., Ramachandran, V., East, A. K., Downie, J. A., and Poole, P. S. (2017). Manganese transport is essential for N_2_-fixation by *Rhizobium leguminosarum* in bacteroids from galegoid but not phaseoloid nodules. *Environ. Microbiol.* 19, e13773. doi: 10.1111/1462-2920.13773

Kovach, M. E., Elzer, P. H., Hill, D. S., Robertson, G. T., Farris, M. A., Roop, R. M., et al. (1995). Four new derivatives of the broad-host-range cloning vector pBBR1MCS, carrying different antibiotic-resistance cassettes. *Gene* 166, 175–176. doi: 10.1016/0378-1119(95)00584-1

Leyva, A., Palacios, J. M., and Ruiz-Argüeso, T. (1987). Conserved plasmid hydrogen-uptake (hup)-specific sequences within Hup*^+^ Rhizobium leguminosarum* strains. *Appl. Environ. Microbiol.* 53, 2539–2543. doi: 10.1128/aem.53.10.2539-2543.1987.

Maunders, E. A., Giles, M. W., Ganio, K., Cunningham, B. A., Bennett-Wood, V., Cole, G. B., et al. (2024). Zinc acquisition and its contribution to *Klebsiella pneumoniae* virulence. *Front. Cell. Infect. Microbiol.* 13, 1322973. doi: 10.3389/fcimb.2023.1322973

Neupane, D. P., Kumar, S., and Yukl, E. T. (2019). Two ABC transporters and a periplasmic metallochaperone participate in zinc acquisition in *Paracoccus denitrificans*. *Biochemistry* 58, 126–136. doi: 10.1021/acs.biochem.8b00854

Patzer, S. I., and Hantke, K. (1998). The ZnuABC high-affinity zinc uptake system and its regulator Zur in *Escherichia coli*. *Mol. Microbiol.* 28, 1199–1210. doi: 10.1046/j.1365-2958.1998.00883.x

Platero, R. A., Jaureguy, M., Battistoni, F. J., and Fabiano, E. R. (2003). Mutations in *sitB* and *sitD* genes affect manganese-growth requirements in *Sinorhizobium meliloti*. *FEMS Microbiol. Lett.* 218, 65–70. doi: 10.1016/S0378-1097(02)01109-6

Saitou, N., and Nei, M. (1987). The neighbor-joining method: a new method for reconstructing phylogenetic trees. *Mol. Biol. Evol.* 4, 406–425. doi: 10.1093/oxfordjournals.molbev.a040454

Schäfer, A., Tauch, A., Jäger, W., Kalinowski, J., Thierbach, G., and Pühler, A. (1994). Small mobilizable multi-purpose cloning vectors derived from the *Escherichia coli* plasmids pK18 and pK19: selection of defined deletions in the chromosome of *Corynebacterium glutamicum*. *Gene* 145, e90324-7. doi: 10.1016/0378-1119(94)90324-7

Simon, R., Priefer, U. B., and Pühler. A. (1983). “Vector plasmids for *in-Vivo* and *in-Vitro* manipulations of Gram-Negative bacteria,” in *Molecular Genetics of the Bacteria-Plant Interaction*, ed. A. Pühler. (Berlin, Springer-Verlagg), 98-106.

Tamura, K., Stecher, G., and Kumar, S. (2021). MEGA11: Molecular Evolutionary Genetics Analysis Version 11. *Mol. Biol. Evol.* 38, 3022–3027. doi: 10.1093/molbev/msab120

Tett, A. J., Rudder, S. J., Bourdès, A., Karunakaran, R., and Poole, P. S. (2012). Regulatable vectors for environmental gene expression in *Alphaproteobacteria*. *Appl. Environ. Microbiol.* 78, e01188-12. doi: 10.1128/AEM.01188-12

Vahling-Armstrong, C. M., Zhou, H., Benyon, L., Morgan, J. K., and Duan, Y. (2012). Two plant bacteria, *S. meliloti* and *Ca.* Liberibacter asiaticus, share functional *znuABC* homologues that encode for a high affinity zinc uptake system. *PLoS One* 7, e37340. doi: 10.1371/journal.pone.0037340

Zhang, P., Zhang, B., Jiao, J., Dai, S. Q., Chen, W. X., and Tian, C. F. (2020). Modulation of symbiotic compatibility by rhizobial zinc starvation machinery. *mBio* 11(2), e03193-19. doi: 10.1128/mBio.03193-19
